# Computational Modeling of Allosteric Communication Reveals Organizing Principles of Mutation-Induced Signaling in ABL and EGFR Kinases

**DOI:** 10.1371/journal.pcbi.1002179

**Published:** 2011-10-06

**Authors:** Anshuman Dixit, Gennady M. Verkhivker

**Affiliations:** 1 Department of Pharmaceutical Chemistry, School of Pharmacy, The University of Kansas, Lawrence, Kansas, United States of America; 2 Department of Pharmacology, University of California San Diego, La Jolla, California, United States of America; National Cancer Institute, United States of America and Tel Aviv University, Israel

## Abstract

The emerging structural information about allosteric kinase complexes and the growing number of allosteric inhibitors call for a systematic strategy to delineate and classify mechanisms of allosteric regulation and long-range communication that control kinase activity. In this work, we have investigated mechanistic aspects of long-range communications in ABL and EGFR kinases based on the results of multiscale simulations of regulatory complexes and computational modeling of signal propagation in proteins. These approaches have been systematically employed to elucidate organizing molecular principles of allosteric signaling in the ABL and EGFR multi-domain regulatory complexes and analyze allosteric signatures of the gate-keeper cancer mutations. We have presented evidence that mechanisms of allosteric activation may have universally evolved in the ABL and EGFR regulatory complexes as a product of a functional cross-talk between the organizing αF-helix and conformationally adaptive αI-helix and αC-helix. These structural elements form a dynamic network of efficiently communicated clusters that may control the long-range interdomain coupling and allosteric activation. The results of this study have unveiled a unifying effect of the gate-keeper cancer mutations as catalysts of kinase activation, leading to the enhanced long-range communication among allosterically coupled segments and stabilization of the active kinase form. The results of this study can reconcile recent experimental studies of allosteric inhibition and long-range cooperativity between binding sites in protein kinases. The presented study offers a novel molecular insight into mechanistic aspects of allosteric kinase signaling and provides a quantitative picture of activation mechanisms in protein kinases at the atomic level.

## Author Summary

Despite recent progress in computational and experimental studies of dynamic regulation in protein kinases, a mechanistic understanding of long-range communication and mechanisms of mutation-induced signaling controlling kinase activity remains largely qualitative. In this study, we have performed a systematic modeling and analysis of allosteric activation in ABL and EGFR kinases at the increasing level of complexity - from catalytic domain to multi-domain regulatory complexes. The results of this study have revealed organizing structural and mechanistic principles of allosteric signaling in protein kinases. Although activation mechanisms in ABL and EGFR kinases have evolved through acquisition of structurally different regulatory complexes, we have found that long-range interdomain communication between common functional segments (αF-helix and αC-helix) may be important for allosteric activation. The results of study have revealed molecular signatures of activating cancer mutations and have shed the light on general mechanistic aspects of mutation-induced signaling in protein kinases. An advanced understanding and further characterization of molecular signatures of kinase mutations may aid in a better rationalization of mutational effects on clinical outcomes and facilitate molecular-based therapeutic strategies to combat kinase mutation-dependent tumorigenesis.

## Introduction

The phenomenon of allosteric communication is fundamental to many biological processes and is recognized as an important factor governing molecular regulation of signal transduction networks [1], [2]. Theoretical and computational studies of allostery in biomolecular systems have witnessed a recent renaissance fueled by the growing interest in the development of quantitative models of allosteric communication in proteins and biological networks [3]–[7]. Sequence-based approaches have unveiled that protein allostery may be mediated by coupled motions of evolutionary conserved yet sparse networks of functional residues which constitute signal communication pathways in proteins [8], [9]. Recent network-based structural studies have also demonstrated that allosteric pathways may be formed through interactions of evolutionary conserved residues that are energetically coupled to mediate long-range communication [10]–[12]. Mechanistic understanding of collective protein motions and allosteric transitions at the molecular level has been significantly advanced by the employment of elastic network models (ENM) and normal mode analysis (NMA) [13]–[25]. These approaches have been further integrated with the information-based Markovian theory of signal propagation [26], [27] and have provided a generalized formalism of allosteric communication in proteins [28]–[31]. Structure-based ENM approaches combined with sequence-based bioinformatics analyses have identified that conserved low-frequency modes of collective motions are robust to sequence variations and capable of transmitting molecular signals over long distances [23]–[25], [31]. Allosteric communication mechanisms can range from a sequential model, where binding of a molecule at one site causes a sequential propagation of conformational changes across the protein, to a fully cooperative model, where structural changes are tightly coupled. More recently, an intermediate, “block-based” model was proposed, where sparse clusters of closely interacting residues can maintain a weak association to other blocks of residues and thus pass information between more distance regions of a protein [32].

Collectively, these studies have shown that allosteric networks orchestrating cooperative protein motions can be formed by evolutionary conserved and sparsely connected groups of residues, suggesting that rapid transmission of allosteric signals through a small network of distantly connected residue clusters may be a universal requirement encoded across protein families. Statistical analyses of motions in allosteric proteins with known inactive and active crystal structures have quantified the magnitude of allosteric effects, revealing a strong preference toward weakly constrained regions such as loops and protein surface regions [33]. Subsequent graph-based analysis and a global communication network model have shown that small-world allosteric networks have sparse connectivity and long-range protein communication is determined by specific residue clusters playing critical roles in the transmission of functional signals [34], [35]. The global communication network (GCN) model has integrated tertiary (residue-scale) and quaternary (subunit-scale) structural changes, providing a more general representation of allosteric communication mechanisms that allowed to simplify atomistic simulations and proven useful in guiding experiments probing allosteric function [35]. Data mining and machine learning methods using support-vector models have helped to infer rules that can distinguish structural hotspots of functionally important allosteric residues [36]. Computational biophysics studies of allosteric regulation have explored a functional linkage between simulations of protein dynamics and energetics of allosteric coupling [37]–[45]. Thermodynamics-based approaches that linked structural perturbations with free energy changes of allosteric coupling have provided quantitative insights into allosteric mechanisms of conformational switching [46]–[49]. A physics-based perturbation method, the Rotamerically Induced Perturbation (RIP), can generate concerted protein motions by applying local torsional perturbations to individual residues [50]. Nonequilibrium methods can monitor concerted protein motions and determine the distribution of signaling pathways, while avoiding long simulation times required in conventional molecular dynamics (MD) simulations [51]. Graph-based analysis of protein allosteric communication can reduce complexity and yield a convenient characterization of the protein architectures as one-dimensional maps comprised of nodes (residues) connected by edges (inter-residue “interactions”) [52]–[61]. These methods have shown that protein structural graphs form small world networks [52]–[55], characterized by high local residue connectivity and a small number of long-range connectivity. Network and graph-based approaches have been employed in predicting protein-protein interactions [54], [55] catalytic sites in enzymes [56], [57], protein structure, energetics and evolution [58]–[61].

The allosteric regulation of protein kinases serves as an efficient strategy for molecular communication and event coupling in signal transduction networks. The regulatory interactions have a major role in determining the conformational dynamics of the kinase domain and activation mechanisms [62]–[69]. Protein kinase regulation may be controlled by a dynamic coupling of two spatially distributed yet conserved and functionally important intermolecular networks between the N-lobe and the C-lobe forming a hydrophobic regulatory spine and a catalytic spine [66]–[69]. The wealth of structure-functional studies about protein kinases has demonstrated that protein kinase activity can be tightly regulated via dynamic interconversion between closely related active and highly specific inactive kinase states - a structural hallmark of the kinase domain which is critical for its normal function [70]–[79]. High-resolution nuclear magnetic resonance (NMR) spectroscopy can complement X-ray crystallography studies by probing protein dynamics on multiple time scales and detecting a site-specific ligand signature that allows differentiation between competitive and allosteric inhibitor binding [80]. NMR studies have detected protein kinase motions in the active and inactive forms on multiple time scales, suggesting that conformational mobility is vital for regulatory control of kinase activity [81]. The dependence of chronic myeloid leukemia (CML) on the translocated BCR-ABL kinase is associated with unique drug responses to small molecule inhibitors [82]. The mechanism of protein kinase regulation via dynamic equilibrium between structurally different functional states has been successfully exploited in the discovery of selective inhibitors targeting inactive conformations of the ABL kinase [83]–[94]. A large number of point mutations that impair the binding of Imatinib (Gleevec) to ABL have been described [83], [84], suggesting that some drug resistant mutations could exist before treatment, and may contribute to tumorigenesis. Structurally conserved gate-keeper mutation ABL-T315I is a dominant cancer-causing alteration, leading to the most severe Imatinib resistance by favoring the active form of the ABL kinase. These findings guided the design of the second-generation ABL inhibitors Dasatinib and Nilotinib [85]–[91]. While these inhibitors are effective against most ABL mutants, the ABL-T315I mutation is still resistant to all three therapies. Most recently, a third-generation of rationally designed analogs and hybrids of Imatinib and Dasatinib, including Ponatinib, DCC-2036 and HG-7-85-01 [92]–[94] were shown to recognize a broad spectrum of inactive kinase conformations and retained potency against ABL-T315I. Nevertheless, activating mutations that destabilize the inactive conformation of ABL (most notably ABL-T315I) still result in reduced binding affinity of these inhibitors. Although the vast majority of protein kinase inhibitors bind to the ATP binding site of the catalytic domain, a considerable effort has been recently invested to discover inhibitors associated with a specific kinase and disease [95]–[100].

Unlike ATP-competitive kinase inhibitors, allosteric inhibitors typically bind outside the catalytic domain and affect kinase activity by eliciting global conformational transformations, which may confer a greater specificity and allow for a subtle modulation of kinase regulation [101]. Allosteric regulation mechanisms in protein kinases may include stabilization of the inactive MEK kinases by targeting the adjacent to the ATP binding pocket in MEK-1, MEK-2 [102], [103] and JNK kinase [104]–[106]; allosteric binding to the myristoyl-binding pocket of ABL and regulation via formation of multidomain ABL-SH2-SH3 complexes [107]–[114]; activation mechanism via formation of regulatory complexes in cyclin-dependent kinase 2 (CDK2) [115], [116], EGFR [117]–[122], HER2/Erb2 [123], HER4/ErbB4 [124], [125]; and allosteric regulation of AKT [126]–[131] and PDK1 kinases [132], [133] via docking of a phosphorylated hydrophobic motif to a hydrophobic pocket on the N-terminal lobe in the catalytic domain. Activation processes in the ABL kinase is linked with the formation of multi-protein regulatory complexes with the SH2 and SH3 domains. Crystallographic studies have determined that in the downregulated inactive state of the ABL-SH2-SH3 complex the SH3-SH2 unit is docked onto the kinase catalytic domain [107], [108]. In contrast, small angle X-ray scattering (SAXS) analysis has detected a dramatic structural rearrangement in the active ABL complex accompanied by the release of the inhibitory interactions and disengagement of the SH2-SH3 domains [107], [108]. Hydrogen exchange mass spectrometry (HX MS) investigation of the ABL-T315I dynamics has provided the first evidence of long-range conformational disturbances caused by activating mutations and allosterically transmitted to the remote protein regions [111]. Recently discovered allosteric inhibitors GNF-2, GNF-5 of the ABL kinase can bind to the myristoyl-binding pocket and independently inhibit kinase activity [112], [113]. HX MS studies of the ABL-T315I dynamics in the presence of ATP competitive inhibitor Dasatinib and GNF-5 have revealed long-range cooperativity between the myristate-binding site and the ATP-binding site induced upon allosteric inhibitor binding that allowed for the effective synergistic inhibition of the ABL-T315I mutant by a drug combination [114].

Structure-functional studies of EGFR kinase domains have revealed that the formation of an asymmetric kinase dimer is critically associated with an activated kinase conformation and is essential for tyrosine kinase activation [117]–[122]. A recent crystal structure of the HER2 kinase domain [123] has provided additional support to allosteric activation via asymmetric dimerization, similar to activation mechanisms in the EGFR and HER4 kinases [124], [125]. In common to the crystal structures of EGFR, HER2 and HER4 kinases, activation mechanisms may exploit an asymmetric head-to-tail dimer, in which the C- lobe of one monomer acts as a “donor” monomer (activator) that interacts with the N-lobe of an adjacent “acceptor” monomer (receiver), stabilizing conformational changes that activate the receiver molecule. Moreover, an asymmetric structural arrangement of a functional EGFR dimer is highly similar to the complex formed by the receiver CDK2 kinase with its activator, cyclin A [115], [116]. Recent studies have shown the importance of the intracellular juxtamembrane EGFR region in promoting activation of an asymmetric dimer via forming a “juxtamembrane latch” between the N-terminal lobe of the receiver and the C-terminal lobe of the activator, allowing to “glue” two kinase monomers and potentiate activation of the receiver molecule [120], [121]. Hence, a unifying structural mechanism associated with asymmetric tyrosine kinase arrangements in regulatory complexes could underlie the activation mechanism of the EGF and ErbB protein families and explain a linkage between ligand-induced receptor dimerization and kinase activation [134]–[137].

Mechanisms of protein kinases regulation have been also studied in computational investigations of c-Src kinase [138]–[145], adenylate kinase [146], ABL kinase [147], CDK5 kinase [148], KIT kinase [149], PKA kinase [150], and AKT/PKB kinase [151], RET and MET kinases [152], [153] and EGFR kinase [154]–[160]. These studies have suggested that functional coupling between collective motions and local structural changes can rationalize the experimental data and provide molecular insights into allosteric mechanisms. We have previously reported that the impact of the gate-keeper mutant on conformational dynamics of ABL may spread far beyond the immediate site of mutation leading to functional changes in conformational mobility at the remote kinase regions [155]. These results corroborated with the HX MS experiments of ABL regulatory complexes [111], pointing to a potential allosteric effect of the activating mutations in the ABL kinase.

Despite recent progress in computational and experimental studies of protein kinase structure and function, the molecular mechanism and dynamics of mutation-induced allosteric kinase activation by regulatory complexes remain mostly qualitative. In this work, we have investigated mechanistic aspects of allosteric activation mechanisms in ABL and EGFR kinases by integrating the results of multiscale simulations with the principal component analysis and computational modeling of signal propagation in proteins. We show that mechanisms of allosteric activation in the ABL and EGFR kinases may be determined by a functional cross-talk between the organizing αF-helix and conformationally adaptive αI-helix and αC-helix. These structural elements form a dynamic network of efficiently communicated clusters that may control the long-range coupling and allosteric activation in the interdomain regulatory complexes. The results of study may reconcile current experimental data pointing to general mechanistic aspects of activating transitions in protein kinases.

## Results/Discussion

### Computational Modeling of Allosteric Communication

In this work, molecular dynamics (MD) simulations, principal component analysis (PCA) and computational modeling of signal propagation in proteins [161], [162] were employed to elucidate molecular principles of allosteric communication in ABL and EGFR kinases and determine allosteric signatures of the gate-keeper cancer mutations at the increasing level of complexity - from catalytic domain (ABL-T315I, EGFR-T790M) to multi-domain regulatory complexes (ABL-T334I, EGFR-T766M). The following specific objectives were pursued in the present study: (a) perform a comparative analysis of the collective protein motions and allosteric communication profiles obtained from simulations of the catalytic domain and regulatory complexes; (b) determine key structural elements and functional residues in ABL and EGFR kinases involved in collective motions and long-range allosteric coupling; (d) analyze and compare long-range communications and allosteric signatures of mutation-induced kinase activation by the gate-keeper mutations in ABL and EGFR. We employed concepts of the absolute and relative Long Range Communication Capability (LRCC) associated with the protein residues in the context of a computational model of signal propagation in proteins [161] (see Materials and Methods for a detailed description).

According to this model, two remote protein residues (or residue clusters) are defined to have a high communication propensity (or communication capability) if the mean-square fluctuation of their inter-residue distance would vary within a relatively small range over long time MD simulations. The higher the fraction of residues that have high communication efficiency with a given residue at an empirically chosen threshold of efficient and fast long-range communications, the greater would be the absolute LRCC of this residue. Under this assumption, a perturbation in one residue in a pair of well-communicated residues should be consistently “communicated” to the “partnering” residue located at a significant distance. Conversely, two residues would not efficiently communicate when the thermal fluctuations of their inter-residue distance would be large and inconsistent with the level of displacements for respective residues, e.g. the inter-residue distance would change in a wide spectrum of values inconsistent with the amplitude of thermal fluctuations of individual residues. This would amount to a slow and inconsistent propagation of a perturbation signal from one residue to the other. A meaningful metric of long-range communication would display fluctuations of the inter-residue distance corresponding and reflecting the order of per residue fluctuations. According to our model, mutational changes can modulate the energy landscape of a protein and alter communication propensities among pairs of residues. The communication propensity histograms of the different protein states scan LRCC that represent the fraction of residues that may have high communication efficiency with a given residue and located at distances greater than a defined threshold from that residue. We hypothesize that the residue clusters characterized by the peaks in the communication efficiency profile may be important for long-range cooperative interactions.

### Allosteric Signatures of Mutation-Induced ABL Activation

In this section, we analyzed collective motions and long-range communications in the ABL kinases using the results of our recently reported simulations of the ABL catalytic domain and multidomain complexes [155] in the following functional states: inactive ABL state (PDB ID 1IEP) [70], the active ABL state (PDB ID 1M52) [71], [72]; the active form of the ABL-T315I mutant (PDB ID 2Z60) [75]; the inactive autoinhibited form of ABL-SH2-SH3 complex (PDB ID 2FO0) and the active form of the ABL complex (PDB ID 1OPL) [108]. For clarity and completeness of the discussion, we summarized our earlier results of 20 ns MD simulations of the ABL complexes [155] in the context of the current objectives (**Figure S1**). According to our results, a structurally stable bundle of α-helices in the C-terminal could be dynamically coupled via regulatory spines with the regions of larger thermal fluctuations corresponded to the P-loop, αC-helix and the activation loop. Moreover, mutation-induced modulation of protein flexibility in the inactive state may be compounded by the increased structural stability of the active form [155].

To better characterize the nature of collective motions between functional kinase regions in regulatory complexes, we analyzed here collective motions of the ABL complexes using PCA of the covariance matrix, calculated from 20 ns MD trajectories of the complete systems (**Figure S2**). The correlation matrix described the linear correlation between any pairs of C_α_ atoms as they move around their average position during simulations. A positive correlation between two atoms could reflect a concerted motion in the same direction, whereas a negative correlation may indicate an opposite direction motion. We noted important similarities and differences between the correlation profiles of inactive (**Figure S2 A**) and active forms (**Figure S2 B**). The covariance map of the active ABL complex displayed an increased and more broadly distributed level of positive correlation, both within the catalytic core, and also between the catalytic domain and the SH2 domain. Overall, the correlated motions along the first eigen mode in the active ABL complex represented a more uniform level of motion between sub-domains with lower amplitude fluctuations as compared to the autoinhibited form. The “breathing” inter-lobe motion of the catalytic core was coupled with the motions of the activation loop, αC-helix and the αG-helix of the C-terminal. The inter-lobe motions were also coupled with the collective inter-domain motions between the catalytic domain and the SH2 domain.

**Figure 1:**
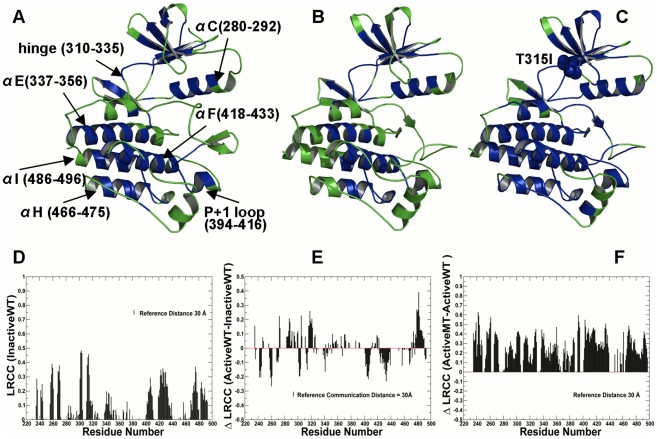
Structural Mapping of Allosteric Communication Profiles in the ABL Kinase Catalytic Domain. Structural mapping of residues involved in long-range communication in different functional states of the ABL catalytic domain: (**A**) the inactive ABL-WT structure (PDB ID 1IEP); (**B**) the active ABL-WT structure (PDB ID 1M52); (**C**) the active form of the ABL-T315I mutant (PDB ID 2Z60). The catalytic core is shown in green. The highlighted in blue allosterically coupled clusters correspond to the peaks in the residue-based LRCC profile computed with the reference communication threshold of 30 Å. The kinase segments and corresponding residue ranges are indicated by respective arrows. (**D**) The absolute LRCC values of the inactive ABL-WT form. (**E**) The relative LRCC values between the active and inactive ABL-WT forms. (**F**) The relative LRCC values between the active ABL-T315I and active ABL-WT. Each bin refers to a residue and shows the fraction of residues that efficiently communicate with this particular residue at distances greater the reference communication threshold of 30 Å.

**Figure 2:**
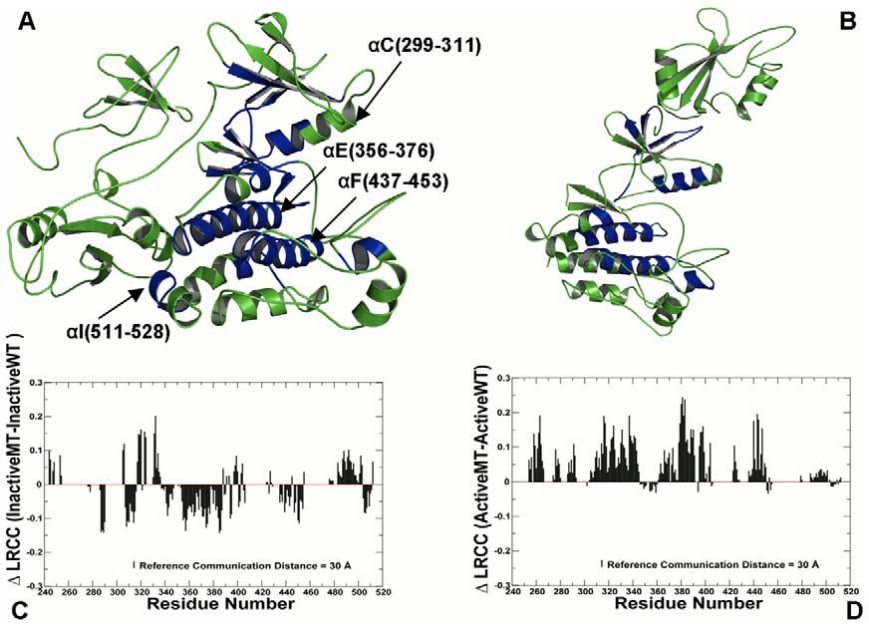
Structural Mapping of Allosteric Communication Profiles in the ABL Regulatory Complexes. The crystal structures of the ABL-SH2-SH3 complex in the inactive, autoinhibited form (PDB ID 2FO0, colored in green) (**A**) and the active form (PDB ID 1OPL, colored in green) (**B**). Structural mapping of the catalytic domain regions involved in the allosteric communication (in blue) correspond to the peaks in the residue-based LRCC profile computed with the reference communication threshold of 30 Å. (**C**) The relative LRCC between the inactive ABL-T334I and the inactive ABL-WT. (**D**) The relative LRCC between the active ABL-T334I and the active ABL-WT.

It is worth noting that the crystal structure of the active ABL complex (PDB ID 1OPL) is completely missing SH3 domain [108]. Consequently, to ensure a consistent comparison of long-range communications between inactive and active complexes, we focused our analysis on the catalytic domain residues and a comparison of their communication profiles derived from simulations of the isolated catalytic core (**Figure 1**) and ABL complexes (**Figure 2**). Structural mapping of long-range communications in the ABL catalytic domain was done using a reference distance of 30 Å (**Figures 1** **A–C**) revealing a coupling between the N-terminal αC-helix (residues 280–292) and a C-terminal core cluster comprised by the αF-helix (residues 418–433), αI-helix (residues 486-496), and αE-helix (residues 337–356) (**Figure 1**). According to our analysis, allosteric communication between a flexible β-strand of the N-terminal lobe, the αC-helix and the P+1 loop could be controlled by the integrating αF-helix. The relative LRCC values between the inactive and active WT forms (**Figures 1** **D–F**) reflected a partial loss in the communication capabilities in the active state and the increased mobility of the αF-helix and the αE-helix, indicative of a more flexible active kinase form. We observed that this effect may be partly offset by a moderate increase in communication propensities of the hinge region (residues 310–335) and the αC-helix (**Figure 1** **D, E**). An interesting finding from this analysis was an appreciable impact of ABL-T315I on long-range communications, manifested in a broadened network of allosterically coupled residues (**Figures 1** **D–F**). This effect could be seen by inspecting changes in the density of allosterically coupled clusters between the inactive states (**Figure 1A**), the active form (**Figure 1B**) **and** the active form of the ABL-T315I mutant (**Figure 1C**). The αC-helix, αF-helix, and αI-helix regained their integrating role in enabling long-range communications in the mutant form. A similar pattern of long-range communications between structurally rigid αF-helix and conformationally adaptive αI-helix, αC-helix and P+1 loop was detected from simulations of the ABL complexes (**Figures 2** **A, B**). The impact of the ABL-T334I mutation on the inactive complex manifested in a decline of the LRCC values and the increased mobility of the αF-helix (residues 437–453), αE-helix (residues 356–376), αI-helix (residues 511–528), and αC-helix (residues 299–311) (**Figures 2** **A,C**). This observation is consistent with MD simulations of ABL complexes [155], where ABL-T315I was shown to weaken the “rigid-clamp” arrangement and destabilize the inactive complex.

The efficient long-range communications between the organizing αF-helix and conformationally adaptive αI-helix and αC-helix is not merely a result of small thermal fluctuations in the respective segments. A dynamic network of long-range interactions between these regions may rather have a functional significance in coordinating collective inter-lobe and inter-domain motions, both of which are known to be important to allosteric communication [35]. A broader and denser network of long-range communicated clusters in the ABL-T315I included also the hinge region, the hydrophobic spine and the catalytically critical Asp-Phe-Gly (DFG) motif from the activation loop (**Figure 1C**). The critical role of the integrated cluster formed by the hydrophobic and catalytic spines, both anchored to the integrated αF-helix, is well recognized as an organizing element regulating protein kinase dynamics and activity [66]–[69]. The cooperative interactions between the αF-helix and the αC-helix may control a dynamic connection between the two lobes of the catalytic core and be important for a dynamic assembly and disassembly of the hydrophobic spine regulating the protein kinase activity. The combined analysis of the correlated motions and long-range communications in the ABL complexes is consistent with a mechanistic model of kinase activation involving cooperative assembly of the hydrophobic spine, the formation of the Src-like intermediate structure, and a cooperative breakage and formation of characteristic salt bridges [155]. It is worth stressing that coupling between rigid and flexible protein regions and correlation of various motions may generally lead to both increases and decreases in thermodynamic stability. A broader network of concerted motions and long-range communications in the mutant form is consistent with our previous finding that all free energy components may act concertedly to enhance the thermodynamic stability of the active ABL-T315I [155].

### A Functional Role of the Interdomain Interface in ABL Activation

**Figure 3:**
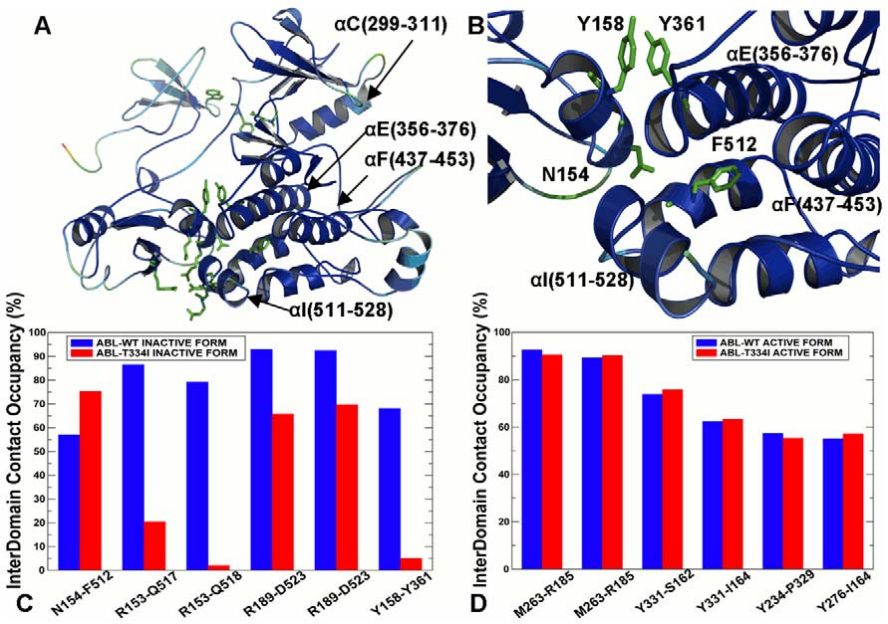
Structure-Functional Analysis of the Inter-Domain Interface in the ABL Regulatory Complexes. (**A**) An overview of the inter-domain interface in the crystal structure of the inactive ABL complex. The crystal structure is colored according to the protein flexibility (light blue corresponds to more flexible regions, dark blue corresponds to structurally rigid regions). The residues involved in the interdomain interface are shown in green sticks. (**B**) A close-up view of the interdomain interface between the kinase catalytic core and the SH2 domain. The critical residues are shown in green sticks. (**C**) The occupancy of the interdomain interface contacts in the inactive, down-regulated form of the ABL-SH2-SH3 complex. (**D**) The occupancy of the interdomain interface contacts in the active form of the ABL-SH2-SH3 complex. The occupancies of the interdomain interface contacts are shown in blue filled bars for the ABL-WT and in red filled bars for the ABL-T334I mutant.

Analysis of long-range communications allowed to highlight a functional role of stabilizing interdomain contacts in the inactive and active ABL complexes (**Figure 3**). In the inactive ABL complex, the SH2 domain is docked closely onto the kinase domain by repositioning and rigidifying the αI-helix of the C-terminal and forming a dense network of hydrogen bonds and packing interactions (**Figure 3** **A,B**). We observed high average occupancies for the major interdomain contacts that maintained their stability throughout a long simulation period. These specific contacts included hydrogen bonding between side-chain of Arg-153 of the SH2 domain and the backbone carbonyls of the kinase domain residues Gln-517 and Glu-518. Additional hydrogen bonding was formed between Arg-189 of the SH2 domain and Asp-523 of the αI-helix. This hydrogen bonding network was further strengthened by packing interactions between Tyr-158 of the SH2 domain, which aromatic ring was perfectly stacked against Tyr-361 from the αE-helix of the kinase domain (**Figure 3** **B**). Importantly, the high occupancies of these interdomain contacts were significantly reduced for the ABL-T334I mutant (**Figure 3** **C**). The interdomain interactions of the active ABL complex included Ile-164 of the SH2 domain interacting with Thr-291 and Tyr-331 of the kinase domain. Interestingly, the occupancies of these core interactions were sustained in the active “top-hat” ABL complex at a relatively high level and even further consolidated for the mutant complex (**Figure 3** **D**).

In agreement with the experimental data, this analysis provided another evidence of a detrimental impact of the activating mutation on structural integrity of the inactive ABL complex that could promote conformational transformation to the active state. Conversely, a stabilizing role of this mutation may be seen in the enhanced structural rigidity of the interface in the active ABL form. Based on this analysis, we could suggest that the activating ABL-T334I mutation could perturb the interdomain interface via allosteric coupling between αF-helix and αI-helix and lead to a significant destabilization of the “rigid-clamp” form of the ABL-SH2-SH3 complex. Hence, the impact of the gate-keeper mutation may be allosterically transmitted to the interdomain regions located at a considerable distance from the mutational site, supporting allosteric nature of the mutation-induced ABL activation. Collectively, these factors could contribute to the mutation-induced allosteric effect that may perturb the thermodynamic equilibrium away from the inactive form towards alternative conformational states and thus serve as a catalyst of activation. These results corroborated with the crystallographic and functional studies of the ABL-T315I mutant [74], [75] confirming an activating nature of the gatekeeper mutation [76]. These findings may have certain relevance in the context of drug resistance effects and design of ABL inhibitors. Our results suggested that the ABL-T315I mutation could allosterically strengthen and coordinate distinctive structural elements of the kinase core, leading to the enhanced structural consolidation of the constitutively active kinase form. As a result, design of ABL inhibitors binding to the active form of the enzyme would inevitably have to overcome competition from cellular ATP. Novel ABL inhibitors of ABL-T315I that bind to the inactive conformation could experience weaker competition from ATP and may act by preventing kinase activation, rather than by inhibiting kinase activity directly.

The evidence of efficient long-range communications in active ABL complexes may be of importance given the rapidly growing interest in developing novel and specific kinase inhibitors inhibition targeting allosteric regions. Indeed, our study may have specific implications in light of recent experimental studies of allosteric kinase inhibition and cooperativity between the myristate- and ATP-binding sites of ABL [113], [114]. HX MS analysis of ABL-T315I in the presence of Dasatinib and allosteric inhibitor GNF-5 demonstrated that binding in the myristate-binding site can elicit allosteric alterations in the conformational dynamics of the C-terminal αI-helix that are propagated to the β-strand of the C-terminal lobe and the ATP-binding site [114]. The analysis of collective motions pointed to a possibility of concerted motions between the β-strand in the N-terminal lobe (residues 260–280 in the inactive complex) and the αI-helix from the C-terminal (residues 446–463 in the inactive complex) (Figure S2). Interestingly, our analysis also suggested that allosteric coupling between a flexible β-strand of the N-terminal lobe, the αC-helix and the P+1 loop may be mediated and controlled by the integrating αF-helix. Moreover, we found that the C-terminal αI-helix and the β-strand of the N-terminal lobe could be involved in the long-range communication of the down-regulated ABL complex and allosteric coupling of these functionally important binding sites could be modulated by the gate-keeper mutation. The importance of these results may be appreciated in the context of experimentally detected allosteric effect of the GNF-5 inhibitor that binds to the myristate-binding site and can allosterically affect the thermodynamic stability of the ATP-binding site residues from the β-strand [113], [114]. Hence, our results are in accordance with a mechanistic view of allosteric ABL activation emerging from the experimental data. We propose that allosteric inhibitor binding with ABL-T315I may lead to concerted changes of conformational mobility in these regions, thereby restoring structural arrangement of the ATP-binding site compatible with Dasatinib binding. A detailed analysis of allosteric ABL inhibition by small molecules is being currently pursued in conjunction with the experimental verification by our collaborators, which a subject of a separate investigation that extends beyond the scope of the current study and will be presented elsewhere.

### Allosteric Signatures of Mutation-Induced EGFR Activation

**Figure 4:**
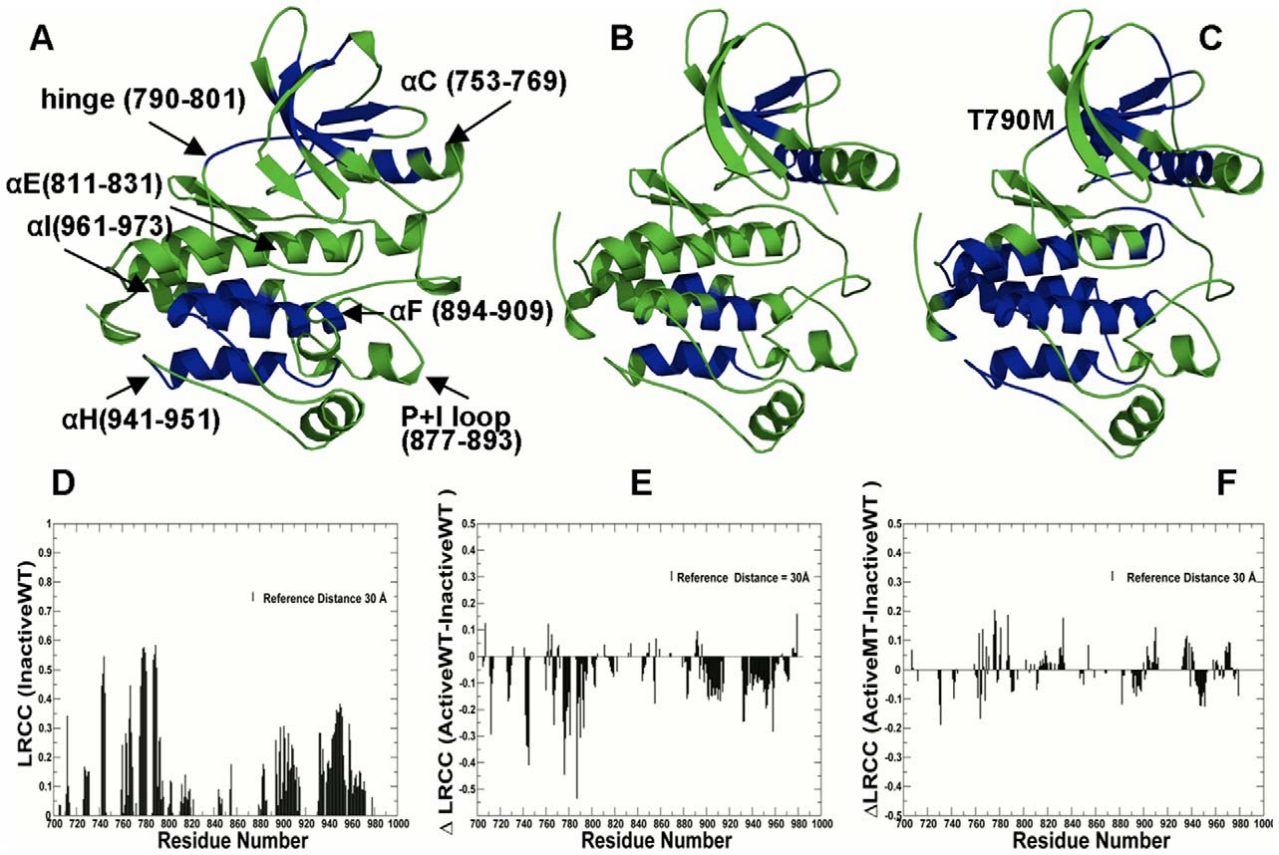
Structural Mapping of Allosteric Communication Profiles in the EGFR Kinase Catalytic Domain. Structural mapping of residues involved in long-range communication in different functional states of the EGFR catalytic domain: (**A**) the inactive EGFR-WT structure (PDB ID 1XKK); (**B**) the active EGFR-WT structure (PDB ID 2J6M); (**C**) the active form of the EGFR-T790M mutant (PDB ID 2JIT). The catalytic core is shown in green. The highlighted in blue are allosterically coupled clusters correspond to the peaks in the LRCC profile computed with the reference communication threshold of 30 Å. The kinase segments and corresponding residue ranges are indicated by respective arrows. (**D**) The absolute LRCC values of the inactive ABL-WT form. (**E**) The relative LRCC values between the active and inactive EGFR-WT forms. (**F**) The relative LRCC values between the active EGFR-T790M mutant and inactive EGFR-WT forms.

In this section, we analyzed allosteric signatures of the EGFR kinase catalytic domain using the results of MD simulations in the following functional states: the inactive EGFR form (PDB ID 1XKK) [77]; the active EGFR form (PDB ID 2J6M) [78], the active form of the EGFR-T790M mutant (PDB ID 2JIT) [79]. The analysis of long-range communications the EGFR catalytic domain revealed similar coupling between structurally rigid αF-helix and conformationally adaptive αI-helix, αC-helix of the catalytic core (**Figure 4**). This effect was seen from inspecting changes in the distribution of communicated residue clusters in the inactive state (**Figure 4A**), the active form (**Figure 4B**) **and** the active form of the EGFR-T790M mutant (**Figure 4C**). Mutation-induced amplification of protein flexibility in the inactive state could be accompanied by the counter-effect of restoring structural stability of the active mutant form (**Figures 4** **D-F**). We found that structural elements of the catalytic core involved in long-range communications may be common in ABL and EGFR, e.g. structural architecture of the kinase fold could determine the basic topology of cooperative interaction network. It is well recognized that the ‘on–off’ equilibrium between the inactive and active EGFR states can be altered by activating mutations, resulting in a net increase in kinase activity. Crystallographic studies have proposed that this equilibrium shift may be a result of structural alterations induced by activating mutations [77]–[79]. Our data support these conjectures by showing that the gate-keeper mutation may allosterically enhance protein mobility in the inactive state and then restore structural integrity of the activated form. This result may be of interest in rationalizing the existing mechanisms of the EGFR-T790M resistance that can substantially suppress the inhibitory effects of EGFR-based drugs Erlotinib and Gefitinib in the treatment of lung cancer [163], [164]. Importantly, this mutation can promote oncogenic activation, uncontrolled cell proliferation and tumorigenesis even in the absence of the selective pressure from the kinase inhibitors. In fact, a recent study showed that the EGFR-T790M harboring resistant clones may be found even in untreated lung cancers [165]. Two different molecular mechanisms were offered to explain how EGFR-T790M could confer drug resistance. Initially, it was proposed that the gate-keeper mutation may detrimentally alter the topology of the ATP-binding pocket that would prevent binding of reversible EGFR inhibitors due to steric hindrance [163], [164]. A number of recently discovered irreversible EGFR inhibitors BIBW2992 [166], PF00299804 [167], and HKI-272 [168], [169] could still inhibit the T790M mutants via covalent binding at the catalytic pocket of EGFR, which was at odds with the steric hindrance mechanism. Another study revealed that EGFR-T790M could increase the ATP affinity back to the EGFR-WT level, which may lead to a reduced potency of any ATP-competitive agent [79], [170]. According to this report, the increased ATP affinity may be a primary mechanism by which EGFR-T790M could confer drug resistance. Furthermore, it was suggested that irreversible binding may not be required for effective inhibition of the T790M mutant [170]. A novel reversible EGFR inhibitor XL-647 can bind EGFR-T790M mutant with an affinity sufficient to compete with ATP [171]. Our results are in line with these studies suggesting that the restored long-range communications and reacquired structural rigidity of the EGFR-T790M mutant may prompt the increased ATP affinity towards this mutant and related drug resistance effects.

### Molecular Dynamics Simulations and PCA of the EGFR Dimers

The initial investigations indicated a potential “negative” impact of the activating mutation on conformational dynamics in the symmetric dimer form [154], [155]. We expanded previous studies and report here the results of 20 ns MD simulations based on the crystal structures of asymmetric and symmetric EGFR dimers (PDB ID 2GS6) in the normal and oncogenic states [117]. The most recent crystal structures of the EGFR kinase domain revealed binding of the extended juxtamembrane latch of the receiver kinase to the activator kinase [120], [121]. 20 ns MD simulations were also performed using the extended crystal structure of a symmetric EGFR dimer PDB ID 3GT8 [120]. The residues 669–682 of the JM-B segment were crystallographically resolved under the same conditions in the crystal structures of both asymmetric and symmetric dimers [117]. For clarity, the analysis focused on MD simulations of these asymmetric and symmetric dimers that included important for activation residues of the JM-B motif. We set out to determine the effect of EGFR-T766M mutant on conformational dynamics of regulatory dimer complexes and to understand the molecular basis of mutation-induced allosteric activation in the functional asymmetric dimer.

**Figure 5:**
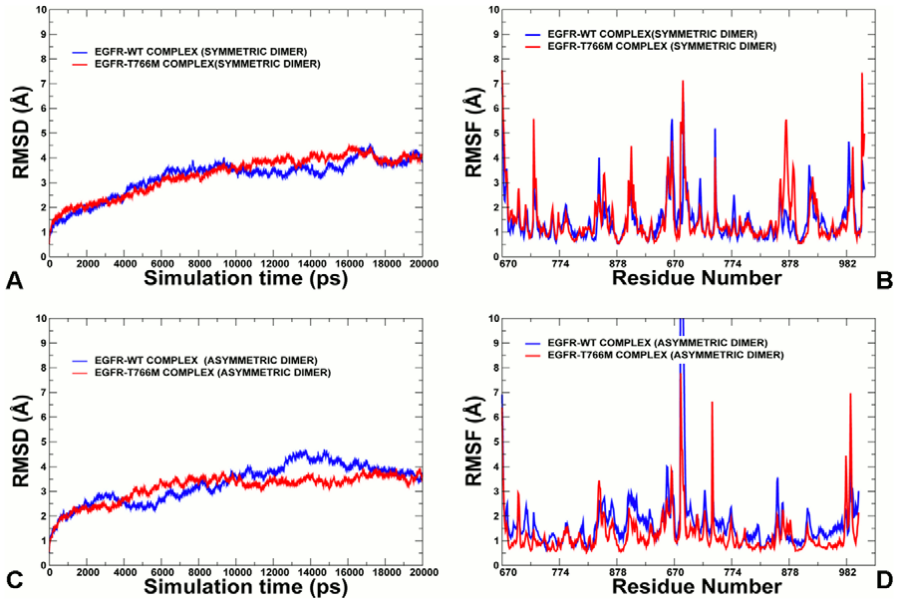
MD Simulations of the EGFR Regulatory Dimers. 20 ns MD simulations were performed for both a symmetric EGFR and asymmetric dimers in the WT and mutant forms. **Upper Panel**: The RMSD fluctuations of the Cα atoms (**A**) and the RMSF values of the Cα atoms (**B**) obtained from MD simulations of a symmetric EGFR dimer (EGFR-WT shown in blue, EGFR-T766M shown in red). **Lower Panel**: The RMSD fluctuations of the Cα atoms (**A**) and the RMSF values of the Cα atoms (**B**) obtained from MD simulations of an asymmetric active EGFR dimer (EGFR-WT shown in blue, EGFR-T766M shown in red).

A comparative analysis of conformational mobility demonstrated an increased structural integrity and a greater stability of an asymmetric dimer that could be enhanced by the activating mutation (**Figure 5**). While the mutational effect in a symmetric dimer led to the increased flexibility as evident from root mean square deviation (RMSD) values, a reduction in thermal fluctuations of the mutant form was seen for an asymmetric EGFR dimer (**Figures 5** **A,C**). Protein flexibility variations were also computed from the root mean square fluctuation (RMSF) of the backbone residues (**Figures 5** **B, D**). The RMSF profiles showed a higher degree of structural variations upon activating mutation in a symmetric dimer, reflected the increased mobility of the activation loop with RMSF  = 5 Å in the mutant as compared to RMSF = 1.5 Å–2 Å for EGFR-WT. Other regions of the enhanced mobility corresponded to the αI-helix at the C-terminal part of the two monomers. In contrast, a global reduction of the conformational mobility extended beyond the immediate site of mutation for an asymmetric EGFR dimer, suggesting that the gate-keeper mutation may allosterically strengthen structural integrity of the functional EGFR form.

The effect of the activating mutation could be further illustrated by monitoring differences in the conformational mobility for two important regulatory elements: the activation loop and the αC-helix (**Figure S3**). Smaller thermal fluctuations of the activation loop were seen not only in the monomer A, that occupies a critical part of the interdomain interface, (**Figure S3 A**), but also in the monomer B, which is located away from the mutational site and the inter-domain interface (**Figure S3 B**). The intrinsic mobility of the αC-helix is critically important in the activation mechanism as one of the central mediators of allosteric changes [117]–[122]. The αC-helix of the receiver monomer A is a key component of the inter-monomer interface in the functional asymmetric dimer. A reduction of the thermal motions in this segment was observed on a longer time scale for both monomers of the asymmetric dimer. Nevertheless, there still remained a certain level of residual mobility in the αC-helix from the activator monomer (**Figure S3 C, D**). In contrast, simulations of a symmetric dimer indicated that the αC-helix of the receiver and the αH-helix of the activator could be more mobile on a longer time scale. These results also agreed with recent computer simulations of ErbB family kinases [158]–[160]. Additionally, structural conservation of the critical salt bridge Glu738-Lys721 plays an important role in kinase regulation [117]. This characteristic salt bridge is fully intact in both inactive and active EGFR conformations, and could only break briefly during the transformation between functional states [155]. A stable behavior of this critical salt bridge was observed in both WT and mutant forms of the asymmetric dimer (**Figure S4**). The mutation-induced stabilization effect was especially pronounced in the monomer B of an asymmetric EGFR dimer (**Figure S4 B**). The dynamics of the inter-monomer interface in the asymmetric dimer may be also controlled by motions of the juxtamembrane region of the receiver that undergoes moderate thermal fluctuations. In the context of these observations, it may be worth pointing out that the increased flexibility of an asymmetric dimer form of the HER2 kinase could lead to a less stable active conformation as compared to EGFR and the low intrinsic catalytic activity [123]. Overall, these results are in line with the mechanism of allosteric EGFR activation, according to which direct contacts between the C-lobe of activator and the N-lobe of the receiver could destabilize autoinhibitory interactions involving the activation loop of the receiver and, as a result, no phosphorylation may be required for activation [134]–[137].

To highlight the principal motions of the active and inactive EGFR dimers, PCA was performed and identified the most relevant displacements of groups by emphasizing the amplitude and direction of the dominant protein motions, through projection on a subset of principal components (eigenvectors) of the covariance matrix calculated from the MD ensemble **(Figure S5)**. We quantified correlated motions within the same monomer and between two monomers for both inactive and active EGFR dimers. In the active, asymmetric dimer a positive correlation emerged not only within each monomer, but also between the activator and receiver monomers **(Figure S5)**. Conversely, in the symmetric dimer positive correlated motions between monomers could be suppressed. The long-range positive cross-correlations extended beyond the intra-monomer regions and could signal the presence of a more diffuse communication network which would favor concerted rigid body motions in the EGFR asymmetric dimer. We observed that asymmetric dimer motions projected onto the principal eigen vector (PC 1) may be determined by coordinated “breathing-like” motions between the activator and the receiver monomers as rigid bodies. The concerted motions of the monomers were accompanied by the low amplitudes “breathing” motions between N-terminal and C-terminal lobes within the monomers, mostly in the activator molecule. These motions are likely determined by the underlying topology of the catalytic domain fold and involve coordinated moves of the P-loop, αC-helix, and the activation loop. However, the reduction of thermal motions in the asymmetric dimer, most notably in the αC-helix and activation loop of the receiver, resulted in mostly suppressed intra-monomer motions of the receiver. This is consistent with the mechanistic role of the activator that via direct interactions with the N-lobe of the receiver can induce and “lock” the characteristic active conformation in the receiver molecule. Our observations also agreed with recent studies of correlated motions in EGFR [157] and provided additional useful insights concerning hierarchy of functional motions in the EGFR regulatory complexes.

### Organizing Principles of Mutation-Induced Activation in the EGFR Kinase: Allosteric Signatures of the EGFR Dimers

**Figure 6:**
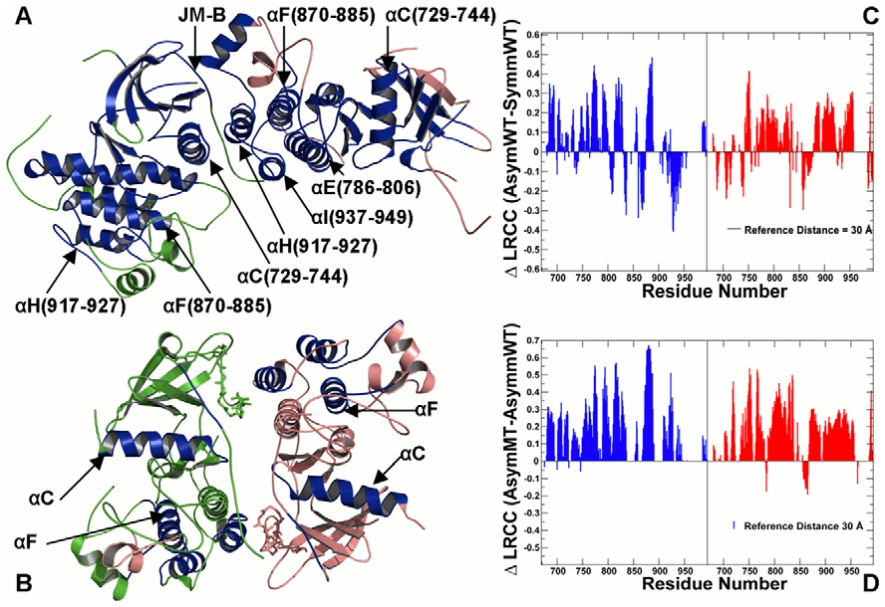
Allosteric signaling in the EGFR Kinase Regulatory Dimers: The Intra-monomer Communications. Structural mapping of the kinase residues involved in the efficient long-range communications at the reference communication distance of 30 Å is shown for an asymmetric dimer (**A**) and a symmetric dimer (**B)**. The reference communication distance of 30 Å was used to analyze primarily long-range intra—domain (intra-monomer) communications and interfacial inter-monomer communications. The depicted mapping and analysis of allosteric communications is based on simulations of the crystal structure of an asymmetric EGFR dimer (PDB ID 2GS6) and inactive symmetric dimer (PDB ID 2GS7). The crystal structures include the monomer A (receiver molecule, colored in green) and the monomer B (activator molecule, colored in pink). The highlighted in blue are allosterically coupled regions. The kinase segments are pointed to by respective arrows. (**C**) The relative LRCC values computed between an asymmetric EGFR-WT dimer and a symmetric EGFR-WT. (**D**) The relative LRCC values computed between an asymmetric EGFR-T766M dimer and a symmetric EGFR-WT. The LRCC values were plotted as respective bars depicted for the monomer A in blue and the monomer B in red.

**Figure 7:**
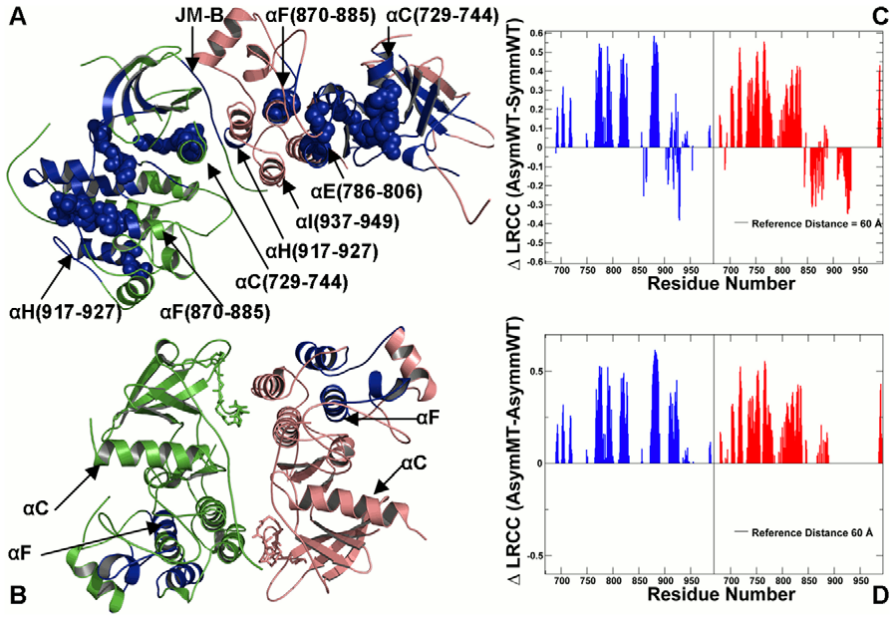
Mutation-Induced Allosteric Signaling in the EGFR Kinase Regulatory Dimers: Analysis of Long-Range Communications between EGFR Monomers. Structural mapping of the kinase residues involved in the efficient long-range communications at the reference communication distance of 60 Å is shown for an asymmetric dimer (**A**) and a symmetric dimer (**B**). We adopted a reference distance of 60 Å to highlight the effect and contribution of the inter-monomer communications. The highlighted in blue are allosterically coupled regions. A schematic representation of allosteric coupling between monomers in an asymmetric EGFR dimer is depicted in blue spheres. The crystal structures include the monomer A (receiver colored in green) and the monomer B (activator colored in pink). (**C**) The relative LRCC values computed between an asymmetric EGFR-WT dimer and a symmetric EGFR-WT (**D**) The relative LRCC values computed between an asymmetric EGFR-T766M dimer and a symmetric EGFR-WT. The LRCC values were plotted as respective bars depicted for the monomer A (receiver molecule, in blue) and the monomer B (activator molecule, in red).

The analysis of correlated motions was supplemented by modeling of long-range communications in the EGFR regulatory dimers at a range of reference distances from 20 Å to 70 Å. For clarity, we focused discussion on two representative cases by making the following assumptions. The reference communication distance of 30 Å was used to analyze primarily long-range intra—domain (intra-monomer) communications and interfacial inter-domain communications **(** **Figure 6**). We adopted a reference distance of 60 Å to highlight the effect and contribution of very long-range inter-monomer communications **(** **Figure 7**). Analysis of long-range communications revealed important attributes that could distinguish active and inactive EGFR dimers. A dense network of long-range inter-monomer communications (reference communication distance of 60 Å) could be seen in an asymmetric dimer (**Figure 7** **A, C**), whereas a sparse network of long-range communications was observed in a symmetric dimer (**Figure 7B**). An asymmetric EGFR-WT dimer could be characterized by the enhanced intra-monomer **(** **Figure 6A, B**) and inter-monomer long-range communications (**Figures 7A, B**) as compared to the symmetric EGFR-WT. Structural mapping revealed the enhanced long-range inter-monomer communications for an asymmetric EGFR dimer (positive ΔLRCC values correspond to improved long-range communication) across a broad spectrum of reference communication distances (**Figure S6**). The introduction of the EGFR-T766M mutation could enhance long-range communications in the asymmetric dimer both at the reference distance of 30 Å **(** **Figure 6** **D**) and 60 Å **(** **Figure 7** **D**). The increased structural integrity of the asymmetric dimer induced by EGFR-T766M could contribute to further stabilization of the active complex. A network of efficiently communicated clusters included the “integrating” αF-helix, the “supporting” αH-helix and the “mediating” αC-helix **(** **Figure 6C**). The inter-monomer coupling is due to stabilizing interactions between the αC-helix of the N-lobe of the receiver and the αH-helix and αI-helices of the C-lobe of the activator. Overall, the combined analysis of correlated motions and long-range communications pointed to the long-range inter-monomer coupling in the asymmetric dimer as an important factor that makes this structural arrangement functionally relevant for activation.

### The αF-Helix and αC-Helix as Integrating and Mediating Elements of Allosteric Coupling

The key structural elements that may control the long-range interdomain coupling and allosteric activation include the “integrating” αF-helix and the “mediating” αC-helix, potentially playing a role of “dispatchers” in regulation of allosteric EGFR activation. Structural analysis indicated that the proper positioning of the αC-helix for activation may be controlled through the αC-β4-loop (744-SVDN-747) immediately following the α-helix [117]–[125]. In both monomers of an asymmetric EGFR dimer, the α-helix could be stabilized via hydrogen-bonding between the main-chain atoms of Ser-744 and Asp-746 of the αC-β4-loop, and the side-chain atoms of Arg-807 and Tyr-803 of the αE-helix in the C-lobe, respectively. We noticed that the residues of the αC-helix and αC-β4-loop were involved in long-range inter-monomer communication. The central interactions stabilizing the inter-monomer interface of an asymmetric dimer are formed by the αC-helix of the receiver and the αI-helix, αH-helix of the activator. These structural elements could also contribute to the network of cooperatively communicating regions that may be strengthened by the activating mutation (**Figures 6** **,** **7**). Structural environment of the αC-helix of the receiver may be a key “mediator” of long-range communications that control allosteric activation of the EGFR dimer. Activation by binding to a hydrophobic patch in the N-lobe of the receiver EGFR interface is a common theme discovered in various crystal structures of an asymmetric dimer [120]–[125]. In contrast, we found that the electrostatically stabilized symmetric dimer may be lacking the effective inter-monomer communication, as the mediating αC-helix was conspicuously absent among long-range interacting regions (**Figure 7C**). This may be a possible mechanistic reason explaining irrelevance of this structural form for activation.

Overall, our results suggested that the effective communication and functional cross-talk between the “integrating” αF-helix and the “mediating” αC-helix, may present an important organizing principle that controls the long-range inter-domain coupling and allosteric activation. The αF-helix along with the hydrophobic and catalytic spines defines the kinase architecture and, together with the αC-helix, may control global motions of the kinase fold. Indeed, all functional motifs in the C-terminal lobe including the activation loop, the catalytic loop, the P+1 loop are anchored to the αF-helix [66]–[69]. We found that the strategic position of the αF-helix may be utilized not only as an integrating scaffold for structural arrangement of other regulatory motifs but also for long-range communications and allosteric activation. Accordingly, using the network-based description of proteins [34], [35], the αF-helix and the αC-helix may be considered as communicating hubs of the regulatory complexes and, as such, may have an impact on allosteric coupling.

### The Juxtamembrane Segment JM-B: A “Stapler” of the Allosteric Network

**Figure 8:**
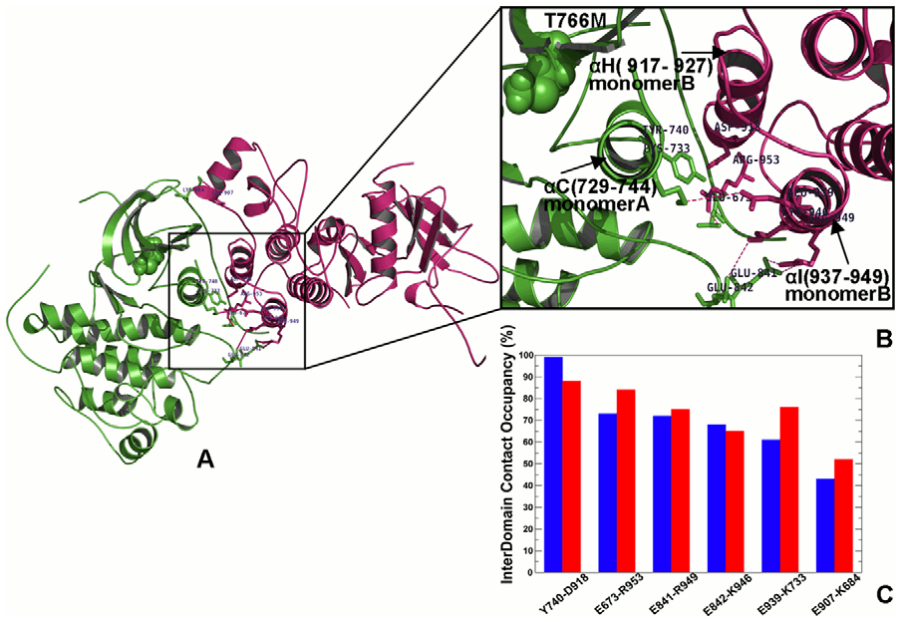
Structural Analysis of the Inter-monomer Interface in Functional Asymmetric EGFR Dimer. (**A**) An overview of the inter-monomer interface in the crystal structure of an asymmetric EGFR dimer (PDB ID 2GS6). The monomer A (receiver molecule) is shown in green and the monomer B (activator molecule) is shown in pink. The mutational site T766M is in green spheres. The interdomain interface is indicated by a rectangular. (**B**) A detailed close-up view of the inter-monomer interface between the N-terminal αC-helix of the receiver monomer A (in green) and the C-terminal αH-helix and αI-helix of the activator monomer B (in pink). (**C**) The occupancy of the critical interdomain interface contacts in an asymmetric EGFR dimer. The occupancies for the EGFR-WT are shown in blue bars and for the EGFR-T766M mutant in red bars.

Recent structural studies showed a critical importance of the “juxtamembrane latch” interactions involving the JM-B segment for activation of the EGFR and HER4 kinase domains [117], [120], [121]. The juxtamembrane segment of human EGFR is formed by the N-terminal half known as the JM-A motif (residues 645 to 663) and the C-terminal half referred as the JM-B (residues 664 to 682) [117]–[125]. However, the nature of allosteric coupling between juxtamembrane segment and the kinase domain is not fully understood. Our simulations and analysis included only the JM-B segment because this region was crystallographically resolved in both the active and inactive EGFR dimers. In an asymmetric dimer arrangement, the activator monomer B makes contacts with the receiver monomer A through interactions involving the αH-helix and αI-helix of the activator as well as the juxtamembrane region and the αC-helix of the receiver (**Figures 7A**, **8A**). We found that the second half of the juxtamembrane segment (JM-B) of the receiver molecule could also contribute to the network of allosterically communicated residues (**Figure 7A**), thus revealing a functionally relevant role of this region in promoting long-range cooperativity and activation of the asymmetric dimer. In addition to the JM-B residues of the receiver molecule, the residues of the αI-helix of the activator interfacing with the JM-B segment were involved in both intra-domain (“short” range cooperativity) and inter-domain communication (“long-range” cooperativity). The recent crystal structures of the EGF receptor and HER4 kinase domains with their juxtamembrane segments have indicated that the JM-B segment could extend from the N-terminal lobe of the receiver to interact with the C-terminal lobe of the activator, thus promoting allosteric activation of the receiver [120], [121]. Our results corroborated with these recent structural studies that pointed to the importance of the juxtamembrane region in activation of an asymmetric dimer. Furthermore, our analysis could offer an additional molecular insight by showing that the juxtamembrane region could facilitate asymmetric dimer formation by assisting the central mediator αC-helix in establishing efficient long-range allosteric communication between monomers. This result was also in agreement with the biochemical experiments that showed that kinase activity of EGFR could be compromised by deletion of the juxtamembrane region [135], [136].

The concerted motions and long-range communications between the activator and receiver molecules could ensure a dynamically enhanced stabilization of the asymmetric dimer required for activation [120], [137]. The JM-B segment of the receiver molecule may act as an “allosteric linker” that could coordinate rigid body motions between the monomers and thus control the dynamics of the dimerization interface critical for activation. These observations are in line with the notion that the juxtamembrane region of EGFR plays a key part in the allosteric activation mechanism by promoting dimerization and further stabilization of the asymmetric dimer [134]–[137]. Our observations are also consistent with the evidence that mutation-based disruption of the electrostatic hook in a symmetric dimer by D979K/E981R and E980R/D982K could reactivate autophosphorylation of EGFR [110], [**124]**–[127****]. An important function of the JM-B segment in modulating mutation-induced EGFR activation may be partly due to its dynamic role acting as the “inter-monomer hinge” during allosteric changes.

According to a recently proposed generalized model of EGFR activation, JM-A segments of both the receiver and the activator may further potentiate asymmetric dimerization and be also required for activation [120], [137], It was proposed that the JM-A segments may form coiled-coil dimer that could further enhance stabilization of the asymmetric dimer and result in activation. Analysis of allosteric communications in the framework of such generalized model of EGFR activation presents a significant computational challenge as it would require currently lacking high-resolution crystallographic information of the complete juxtamembrane region to allow for more accurate biophysical modeling. We currently pursue combined homology modeling and computer simulations of the EGFR complexes involving a complete juxtamembrane region. This investigation extends beyond the scope and focuses of the present work and will be presented elsewhere.

### Allosteric Effect of the Activating Mutation on the Dimerization Interface in EGFR Complexes

The hydrophobic dimerization interface of an asymmetric dimer involves the bottom of the C-lobe of the activator molecule docked on the top of the N-lobe of the receiver molecule (**Figure 8**). We observed that the key residues contributing to the inter-monomer interface could be involved in efficient allosteric communication and the principal interactions between these residues may be allosterically stabilized by the activating mutation (**Figure 8** **A, B**). For instance, we observed a high occupancy of the interfacial interactions between Tyr-740 from the αC-helix (monomer A) and Asp-918 from the αH-helix (monomer B). The effect of the activating mutation could be seen in further strengthening of this contact (**Figure 8** **B, C**). Similarly, the effect of the activating mutation was reflected in further consolidation of the interfacial hydrogen bonding interactions Glu-841--Arg-949 and Glu-842--Arg-946 between the activation loop residues (monomer A) and the αI-helix residues of the monomer B (**Figure 8** **B, C**).

**Figure 9:**
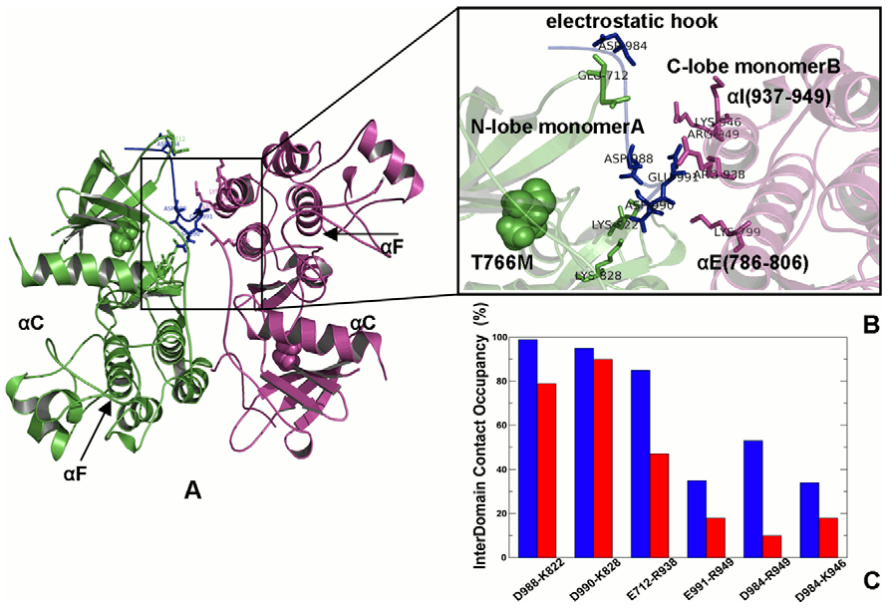
Structural Analysis of the Inter-monomer Interface in a Symmetric EGFR Dimer. (**A**) An overview of the inter-monomer interface in the crystal structure of a symmetric inactive EGFR dimer (PDB ID 2GS7). The monomer A (receiver molecule) is shown in green and the monomer B (activator molecule) is shown in pink. The primary interdomain interface is indicated by a rectangular. (**B**) An exploded view of the electrostatic inter-monomer interface formed between the N-terminal lobe of the receiver monomer A (shown in green), residues from the “electrostatic hook (shown in magenta) and the C-terminal αI-helix and the αE-helix of the activator monomer B (in pink). (**C**) The occupancy of the critical interdomain interface contacts in a symmetric EGFR dimer. The occupancies for the EGFR-WT are shown in blue filled bars and for the EGFR-T766M mutant in red filled bars.

Hence, the effect of the gate-keeper mutation may be allosterically transmitted to the inter-monomer interface residues from the αC-helix (monomer A), αH-helix, and αI-helix (monomer B), supporting allosteric nature of the mutation-induced activation mechanism. Mutation-induced structural stabilization of the interdomain interface coupled with the enhanced long-range cooperativity in a functional asymmetric dimer could rationalize the existing experimental data. Indeed, EGFR activation may be suppressed by mutations in the αH-helix and αI-helix of the monomer B (R938E, I942E, and K946E) that caused the loss of kinase activity [117]. In the head-to-head structure of a symmetric EGFR dimer, two kinase monomers are stabilized by a dense network of salt bridges and hydrogen bonds that connect the kinase monomer through the C-terminal fragments (**Figure 9** **A**) [117], [120]. Based on this crystal structure of a symmetric EGFR dimer (PDB ID 2GS7 [117]), it was initially proposed that the “electrostatic hook” formed between the C-terminal tail (residues 979–990) and the hinge region in the kinase domain may stabilize structural topology of a symmetric dimer (**Figures 9** **A, B**). The inter-monomer interface is formed by Asp-988, Asp-990 from the electrostatic hook, Lys-822 and Lys-828 of the N-terminal of the monomer A and Lys-799, Arg-938, and Lys-946 of the C-terminal lobe of the monomer B.

**Figure 10:**
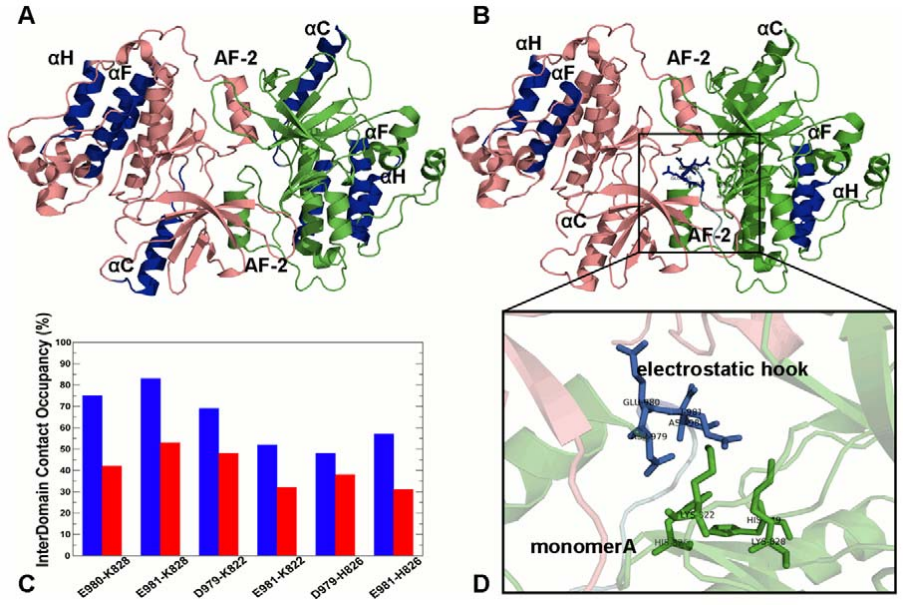
Allosteric Communications and Stabilizing Interactions in an Extended Structure of a Symmetric EGFR Dimer. Structural mapping of the kinase regions involved in long-range communications (highlighted in blue) at the reference communication distance of 30 Å (**A**) and 60 Å (**B)**. The monomer A (receiver molecule) is shown in green and the monomer B (activator molecule) is shown in pink. The extended crystal structure of the inactive symmetric dimer (PDB ID 3GT8) was used in simulations and allosteric analysis. An overview of the inter-monomer interface is indicated by a rectangular in (**B**). (**C**) The occupancy of the critical interdomain interface contacts in the symmetric EGFR dimer. The occupancies for the EGFR-WT are shown in blue bars and for the EGFR-T766M mutant in red bars. (**D**) An exploded view of the electrostatic hook formed between the C-terminal tail residues 979–990 (shown in pink) and the hinge region in the receiver kinase domain (shown in green).

We found that the inter-monomer long-range communication propensities were significantly impaired in this structural form (**Figures 10** **A, B**). Interestingly, the EGFR-T766M mutation resulted in a noticeable reduction of the inter-monomer interaction occupancies, which were mostly determined by the “electrostatic hook” residues (**Figure 9B, C** **)**. Hence, the activating mutation may lead to the weakening of the electrostatic hook, which is a critical “stapling” element protecting the inactive symmetrical dimer. We also analyzed long-range communication profiles obtained from simulations of the most recent crystal structure of a symmetric EGFR dimer that included AF-2 helices in the C-terminal tail (**Figure 10**) [(PDB ID 3GT8 [120]). Similarly, it appeared that the inter-monomer long-range communication (at the reference threshold of 60 Å) was reduced and only the local hydrophobic environment of the αF-helix could contribute to a long-distance cross-talk between monomers (**Figure 10** **A,B**). In this crystal structure the formation of the juxtamembrane latch may be compromised by the “electrostatic hook” and AF-2 helices in the C-terminal tail [120]. The “electrostatic hook,” which is located near the αC/β4 loop of the kinase domain, includes Asp-979, Glu-980, and Glu-981. These residues form salt-bridges with Lys-822, Lys-828, His749, and His-826 of the kinase domain (**Figure 10** **C, D**). The results indicate that in a symmetric dimer arrangement, which is protected by the electrostatic hook residues, the αC-helices could be blocked from establishing long-range communication and, hence, their mediating role in promoting activation could be compromised. This may present a feasible mechanism preventing formation of alternative dimer arrangements [110]. The efficient long-range cooperativity and allosteric communications may be thus an important attribute of the functional regulatory complex.

### Conclusions

We have studied molecular mechanisms of allosteric regulation in the ABL and EGFR protein kinases by integrating multiscale simulations with computational modeling of long-range communications in the regulatory complexes. The results have unveiled organizing principles of mutation-induced activation in the ABL and EGFR kinases that may be orchestrated by a cross-talk between the integrating αF-helix and the mediating αC-helix, responsible for coordination of the inter-domain coupling between key regulatory regions. These findings are in agreement with the central involvement of αF-helix and αC-helix in regulatory functions and allosteric activation. We have shown that collective motions and the efficient inter-monomer communications between the activator and receiver molecules could allow for a dynamically enhanced stabilization of the asymmetric dimer required for activation. Hence, the effective communication between the “integrating” αF-helix and the “mediating” αC-helix may coordinate coupling between the intra-domain and the inter-domain motions and thus be important for allosteric activation. The results of this study have unveiled that structurally conserved gate-keeper mutations may serve as catalysts of kinase activation by increasing long-range communication capabilities and promoting the enhanced stabilization of the active kinase form. We suggest that structural architecture of the regulatory kinase complexes and the intrinsic dynamic equilibrium between major conformational states can define the topology of allosteric networks, while specific communication pathways may be modulated by the mutation or binding partner. The results of our study reconcile recent experimental studies of allosteric kinase mechanisms and provide a useful molecular insight into hierarchy of functional motions and mechanistic aspects of allosteric kinase signaling at the atomic level.

## Materials and Methods

### Structure Preparation

The coordinates of the ABL and EGFR kinase catalytic domain and regulatory complexes in various conformational states were obtained from the Protein Data Bank (PDB) (www.pdb.org) [172]. In MD simulations of the ABL and EGFR kinase domains, we used the following crystal structures : PDB ID 1IEP (inactive ABL structure) [70], PDB ID 1M52 (active ABL structure) [71], [72], PDB ID 2G1T (Src-like inactive ABL structure) [109], PDB ID 2Z60 (the active form of the ABL-T315I mutant) [75], PDB ID 1XKK (Src/Cdk-like inactive EGFR structure) [77], PDB ID 2GS7 (Src/Cdk-like inactive EGFR structure) [109], and PDB ID 2J6M (active EGFR structure) [78], and PDB ID 2JIT (EGFR-T790M mutant) [79]. In MD simulations of the ABL complexes, we employed the crystal structure of the ABL-SH2-SH3 complex in the inactive form (PDB ID 2FO0) and the active form (PDB ID 1OPL) [107], [108]. In MD simulations of the EGFR regulatory complexes we utilized the crystal structures of an asymmetric, active dimer (PDB ID 2GS6 [117]) and inactive symmetric dimers (PDB ID 2GS6 [117], PDB ID 3GT8 [120]). The juxtamembrane segment of human EGFR is formed by the N-terminal half (JM-A motif, residues 645 to 663) and the C-terminal half referred (JM-B motif, residues 664 to 682). The residues 669-682 of the JM-B motif have been determined in the crystal structures of asymmetric and symmetric EGFR dimers [117] and were included in MD simulations and subsequent allosteric communication analysis. We have also analyzed crystal structures of the EGFR asymmetric dimer in the presence of its complete juxtamembrane segment (PDB ID 3GOP [121]), asymmetric dimer of the HER4 kinase (PDB ID 2R4B [119]). All crystallographic water molecules, bound inhibitors, and other heteroatoms were removed. The retrieved structures were examined for missing and disordered residues. The missing residues and unresolved structural segments were modeled using the program MODELLER which is an automated approach to comparative protein structure modeling by satisfaction of spatial restraints [173].

### MD Simulations

MD simulations of the EGFR regulatory complexes (each of 20 ns duration) were performed from the crystal structures of an asymmetric and symmetric dimers (PDB ID 2GS6 [117], PDB ID 3GT8 [120]). MD simulations were carried out using NAMD 2.6 [174] with the CHARMM27 force field [175], [176] and the explicit TIP3P water model as implemented in NAMD 2.6 [177]. The VMD program was used for the preparation and analysis of simulations [178]. The employed MD protocol was described in full details in our earlier study [155]. In brief, the EGFR dimers were solvated in a water box with the buffering distance of 10 Å. Assuming normal charge states of ionizable groups corresponding to pH 7, 39 sodium (Na^+^) and 23 chloride (Cl^−^) counter-ions at physiological concentration of 0.15 mol/L were added to achieve charge neutrality in MD simulations of the asymmetric EGFR dimer. 33 Na^+^ and 17 Cl^−^ counter-ions were added in MD simulations of the symmetric EGFR dimer. All Na^+^ and Cl^−^ ions were placed more than 8 Å away from any protein atoms and from each other. Equilibration was done by gradually increasing the system temperature in steps of 20K starting from 10K until 310K and at each step 10,000 steps of equilibration was run keeping a restraint of 10 Kcal mol^−1^ Å^−2^ on the protein alpha carbons (C_α_). Thereafter the system was equilibrated for 150,000 steps at 310K (NVT) and then for further 150,000 steps at 310K using Langevin piston (NPT) to maintain the pressure. Finally the restrains were removed and the system was equilibrated for 500,000 steps to prepare the system for simulation.

An NPT simulation was run on the equilibrated structure for 20 ns keeping the temperature at 310K and pressure at 1 bar using Langevin piston coupling algorithm. The integration time step of the simulations was set to 2.0 fs. The SHAKE algorithm was used to constrain the lengths of all chemical bonds involving hydrogen atoms at their equilibrium values and the water geometry was restrained rigid by using the SETTLE algorithm. Nonbonded van der Waals interactions were treated by using a switching function at 10 Å and reaching zero at a distance of 12 Å. The particle-mesh Ewald algorithm (PME) as implied in NAMD was used to handle long range electrostatic forces.

### Essential Dynamics and Principal Component Analysis

The covariance matrix between residues i and j represented by the 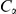 atoms was calculated for each of the 20 ns MD simulation trajectories by averaging motions of 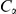
atoms deviating from the mean structure. A total of 500 snapshots from were taken from trajectories and only 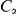 was used for analysis. Translational and rotational degrees of freedom are eliminated and the average atomic coordinates,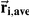 i = 1,..., N, are calculated along the MD trajectory [161]. Essential dynamics (ED) analysis reduces the dimensionality of the covariance matrix by diagonalization. This method describes global protein motions that are represented by the matrix eigenvectors and eigen values. The essential directions of correlated motions during dynamics are calculated by diagonalizing the covariance matrix 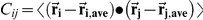.

The correlation matrix 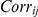 represents the correlation between the motion of atom i and of atom j, obtained from the reduction and normalization of the covariance matrix.



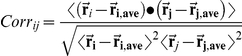



The eigenvectors represent the directions in the multidimensional space that correspond to independent modes of atomic motion, while the eigen values represent their corresponding amplitudes [179], [180]. The magnitudes of eigenvectors are represented by their eigen values and the principal components of protein motions are analyzed by projecting MD trajectories onto directions corresponding to the largest eigen vectors. The correlation value is the normalized covariance matrix, ranging from −1 to 1. Since PCA makes it possible to identify amplitude and direction of relevant protein motions, the projections of the first principal component (PC1) have been analyzed. The calculations were performed using the CARMA package [181] and PCA_NEST web-based service [182].

### Modeling of Long-Range Communications

Signal propagation and inter-domain communication events in proteins can be linked to the fluctuation dynamics of atoms, defining the communication propensity (CP) of a pair of residues as a function of the fluctuations of an inter-residue distance. To evaluate communications between protein residues, we computed a communication propensity 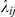 between two residues 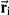 and 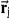 that can be defined as the mean-square fluctuation of the inter-residue distance 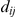:
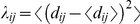
where 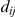 is the distance between the Cα atoms of residue i and residue j.

We investigate the modulation of long-range communication propensities as a function of activation mutation using the theoretical approach previously developed for the analysis of signal propagation [161]. To determine whether a communication between two residues is efficient, we introduced the communication threshold 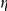, an important parameter in our analysis that is considering only 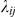 values associated with close amino acids. To determine the value of communication threshold, for each residue i we considered only its communication propensities with the neighbor residues between i-4 and i+4:
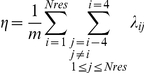
where m is the total number of terms taken into account in the sum; N_res_ is the total number of residues. Therefore, 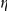 represents the average communication capability between nearby protein residues and, assuming that such residues communicate well, we propose to exploit this quantity as a measure to assess if two spatially separated amino acids in the protein can communicate effectively. Exploiting this threshold definition, we say that a couple of residues efficiently communicate if and only if their communication propensity 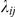 is lower or equal to 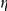. The computed average communication capability between nearby protein residues was about 0.025. We set this value as the threshold for discriminating fast communications.

By considering a given reference distance δ, we define LRCC as the density 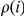 for a given residue i obtained from the fraction of residues that efficiently communicate with it (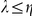) at distances larger than δ. The higher is the fraction of such residues, the higher is the capability of residue R_i_ to establish efficient long-range communications. This quantity simplifies analysis of the communication propensity calculations and allows to readily monitor changes in the communication capabilities of the residues upon mutations. It is helpful to consider a difference between Absolute LRCC values associated with two different simulations and obtain Relative Long Range Communication Capabilities (designated in the Results/Discussion as ΔLRCC). We typically consider the LRCC values associated with a simulation of the WT protein kinase as a reference. The resulting relative measure may highlight mutation-induced differences in communication patterns of the two proteins. The positive ΔLRCC values between simulations of the mutant and WT systems correspond to the enhanced long-range communications in the mutant.

To evaluate signal communications between protein residues in the ABL and EGFR catalytic domains, we have computed the communication propensity 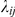 between all protein residues 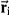 and 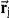
_._ Exploiting a spectrum of reference threshold distances (20 Å, 30 Å), we have then computed the absolute Long Range Communication Capability (LRCC) for each protein residue in the catalytic domain, that is defined as a fraction of residues that efficiently communicate with it (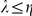) at distances larger than δ = 20 or 30 Å respectively. MD simulations of EGFR dimers employed the crystal structures with missing loop residues between 724 and 726, and from 968 and 980. Additionally, MD simulations of a symmetric dimer considered two (one per monomer) additional five-residue loops (E-I-Y-G-E) that were structurally present yet disconnected from the rest of the protein system in the crystal structures. For the sake of clarity, these loops were omitted in the allosteric communication analyses. For each simulation taken into account, we determined the communication propensity between all the protein residues (excluding the five-residue loops in the “symmetric” dimer) and then computed Absolute LRCC with the reference distance of 20, 30, 40, 50, 60 and 70 Å. It is important to note that we used 20, 30 and 40 Å as a reference distance to analyze long-range intra--domains communications (or simply, long-range communications) whereas we adopted 50, 60 and 70 Å as a reference distance to highlight the effect and contribution of long-range inter--domains communications (or simply, very long-range communications). The resulting histograms allowed to scan communication efficiencies, where each bin refers to a residue and gives the fraction of residues that have high communication efficiency with it at distances larger than the cutoff.

## Supporting Information

Figure S1 **Overview of MD Simulations of the ABL-SH2-SH3 Regulatory Complexes.** 20 ns MD simulations were performed for the inactive, autoinhibited form and active forms. **Upper Panel**: The RMSD fluctuations of the Cα atoms (**A**) and the RMSF values of the Cα atoms (**B**) obtained from MD simulations of the inactive, autoinhibited form of ABL-SH2-SH3 complex (PDB ID 2FO0). ABL-WT shown in blue, ABL-T334I shown in red. **Lower Panel**: The RMSD fluctuations of the Cα atoms (**A**) and the RMSF values of the Cα atoms (**B**) obtained from MD simulations of the active ABL form (“top-hat”) (PDB ID1OPL). ABL-WT shown in blue, ABL-T334I shown in red. Note the crystal structure of the active ABL complex (PDB ID 1OPL) is completely missing SH3 domain [108].
(TIF)

Figure S2 **PCA Analysis of ABL Complexes.** The covariance matrix were calculated from 20 ns MD trajectories of the complete ABL-WT complexes in the downregulated, autoinhibited form (**A**) and active, “top-hat” form (**B**). Translational and rotational degrees of freedom are eliminated and the average atomic coordinates are calculated using 500 frames from the 20 ns MD trajectories. The essential directions of correlated motions during dynamics were then calculated by diagonalizing the covariance matrix *C_ij_*. MD trajectories were projected onto the main essential direction, corresponding to the largest eigenvector. A positive correlation close to 1 (color code red) corresponds to highly coordinated motion of the residue pair along the same direction, whereas a negative correlation (color code blue) indicates motion in opposite directions.
(TIF)

Figure S3 **Protein flexibility of the EGFR catalytic core regulatory regions. Upper Panel:** The RMSD fluctuations of Cα atoms in the activation loop of the monomer A (upper left panel A) and monomer B (upper right panel B) of an asymmetric EGFR dimer. EGFR-WT RMSD values shown in blue and EGFR-T766M shown in red. **Lower Panel:** The RMSD fluctuations of Cα atoms in the αC-helix of the monomer A (lower left panel C) and monomer B (lower right panel D) of an asymmetric EGFR dimer. EGFR-WT RMSD values shown in blue and EGFR-T766M shown in red.
(TIF)

Figure S4 **Time-dependent history of salt bridges from MD simulations of an asymmetric EGFR dimer.** Thermal fluctuations of the salt bridge Glu738-Lys721 of the monomer A **(left panel A**) and monomer B (**right panel B)** of an asymmetric EGFR dimer. The flexibility profiles for EGFR-WT shown in blue and EGFR-T766M shown in red.
(TIF)

Figure S5 **PCA Analysis of EGFR Dimers.** The covariance matrix was calculated from 20 ns MD trajectories of the EGFR-WT asymmetric (**A**) and symmetric dimers (**B**). Translational and rotational degrees of freedom are eliminated and the average atomic coordinates are calculated using 500 frames from the 20 ns MD trajectories. MD trajectories were projected onto the main essential direction, corresponding to the largest eigenvector. Cross-correlations of residue-based fluctuations vary between +1 (fully correlated motion; fluctuation vectors in the same direction, colored in dark red) and −1 (fully anti-correlated motions; fluctuation vectors in the same direction, colored in dark blue). Here, the values above 0.5 are colored in dark red and the lower bound in the color bar indicates the value of the most anti-correlated pairs.
(TIF)

Figure S6
**The Relative LRCC profiles for the EGFR Regulatory Dimers.** The relative LRCC values between the asymmetric and symmetric EGFR-WT dimers computed at a range of reference communication distances: 20 Å (**A**), 40 Å (**B**), 50 Å (**C**), and 70 Å (**D**). Each bin refers to a residue and shows the fraction of residues that efficiently communicate with it (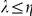) at distances greater the reference communication threshold of 30 Å.(TIF)
