# Supplementary figures and images for: Computational Modeling of Allosteric Communication Reveals Organizing Principles of Mutation-Induced Signaling in ABL and EGFR Kinases
Source: PLoS Comput Biol. 2011 Oct 6;7(10):e1002179. doi: 10.1371/journal.pcbi.1002179 (PMC3188506; doi:10.1371/journal.pcbi.1002179)

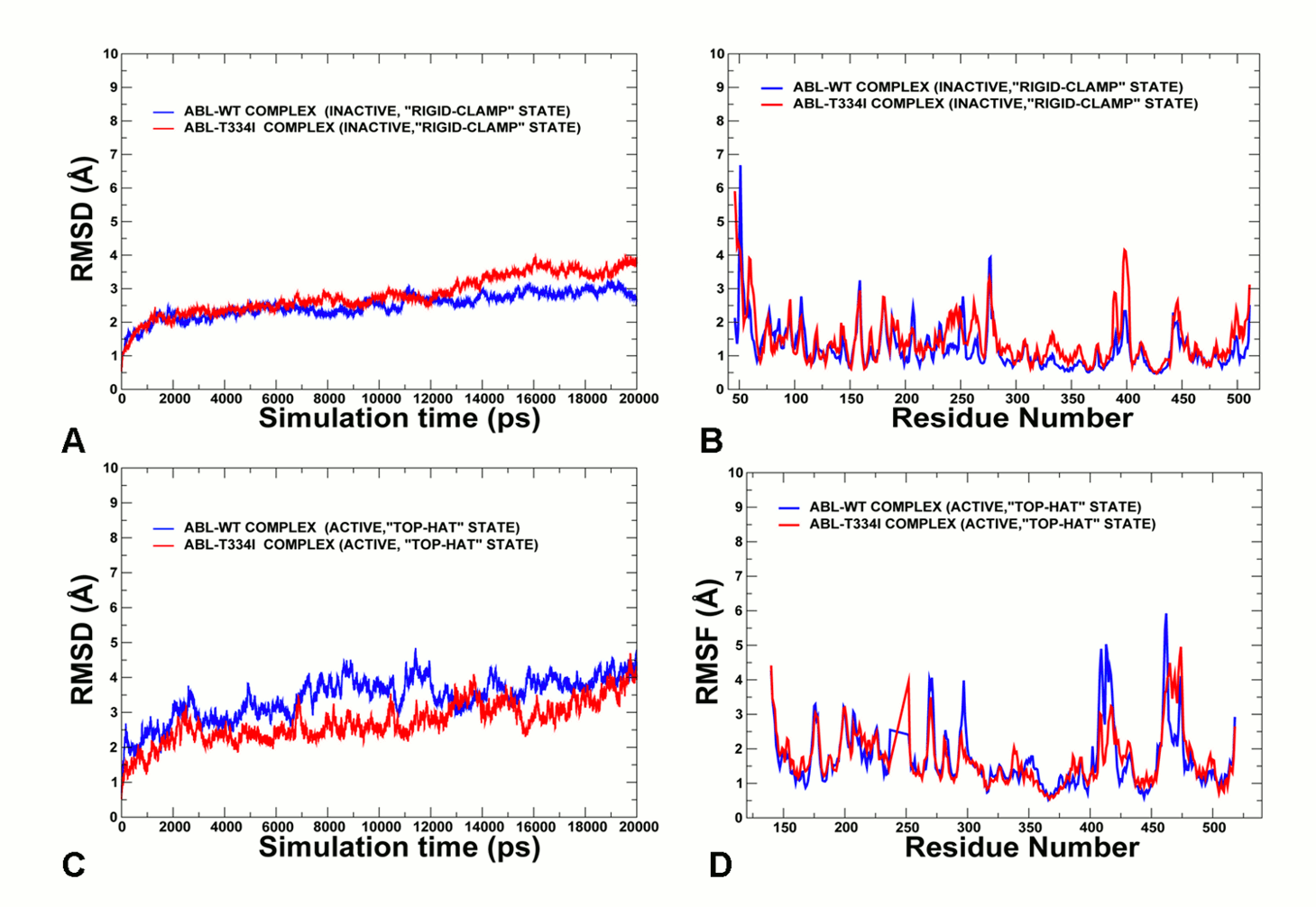

Supplement: Figure S1 — Overview of MD Simulations of the ABL-SH2-SH3 Regulatory Complexes. 20 ns MD simulations were performed for the inactive, autoinhibited form and active forms. Upper Panel: The RMSD fluctuations of the Cα atoms (A) and the RMSF values of the Cα atoms (B) obtained from MD simulations of the inactive, autoinhibited form of ABL-SH2-SH3 complex (PDB ID 2FO0). ABL-WT shown in blue, ABL-T334I shown in red. Lower Panel: The RMSD fluctuations of the Cα atoms (A) and the RMSF values of the Cα atoms (B) obtained from MD simulations of the active ABL form (“top-hat”) (PDB ID1OPL). ABL-WT shown in blue, ABL-T334I shown in red. Note the crystal structure of the active ABL complex (PDB ID 1OPL) is completely missing SH3 domain [108]. (TIF) [file pcbi.1002179.s001.tif]

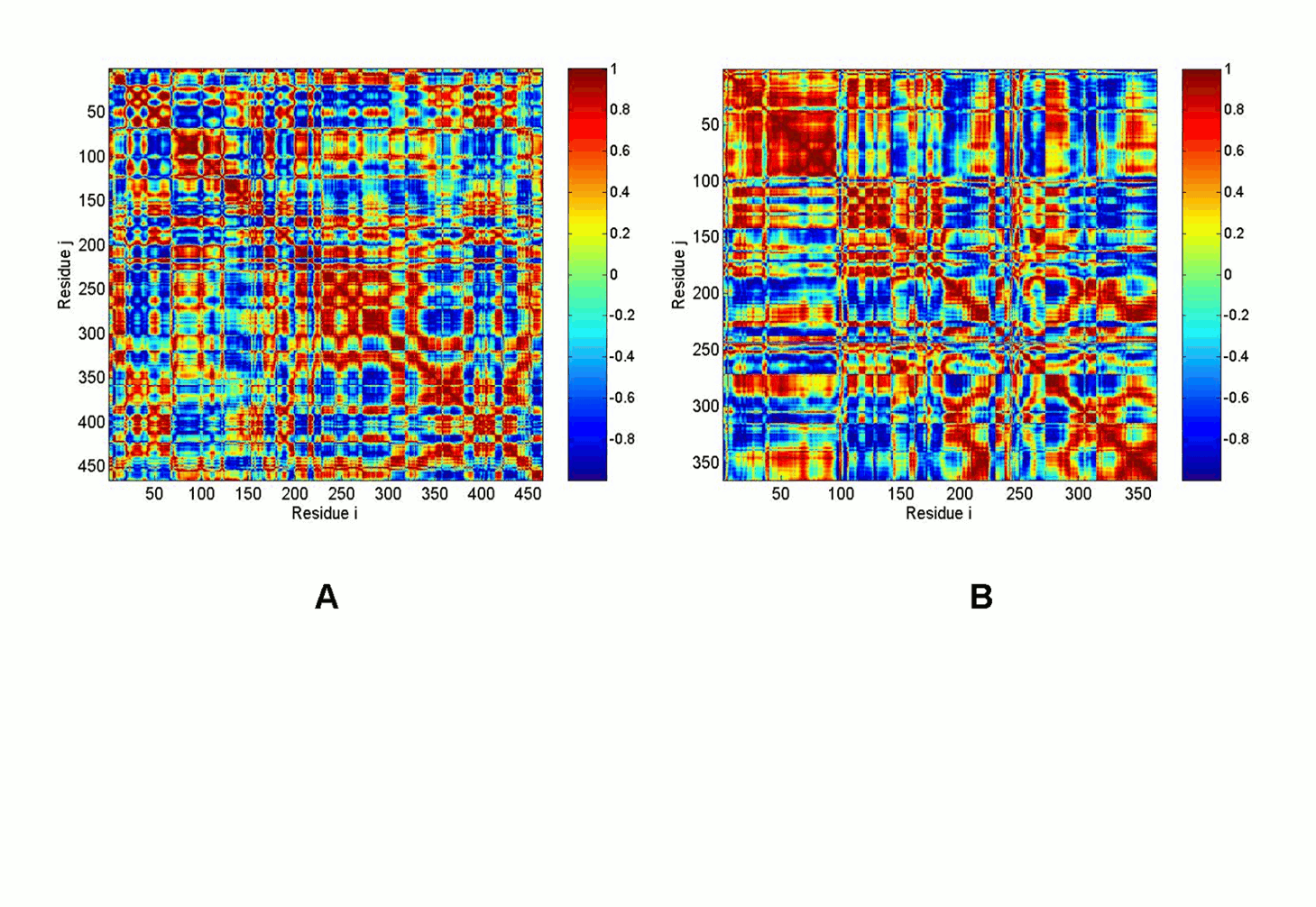

Supplement: Figure S2 — PCA Analysis of ABL Complexes. The covariance matrix were calculated from 20 ns MD trajectories of the complete ABL-WT complexes in the downregulated, autoinhibited form (A) and active, “top-hat” form (B). Translational and rotational degrees of freedom are eliminated and the average atomic coordinates are calculated using 500 frames from the 20 ns MD trajectories. The essential directions of correlated motions during dynamics were then calculated by diagonalizing the covariance matrix Cij. MD trajectories were projected onto the main essential direction, corresponding to the largest eigenvector. A positive correlation close to 1 (color code red) corresponds to highly coordinated motion of the residue pair along the same direction, whereas a negative correlation (color code blue) indicates motion in opposite directions. (TIF) [file pcbi.1002179.s002.tif]

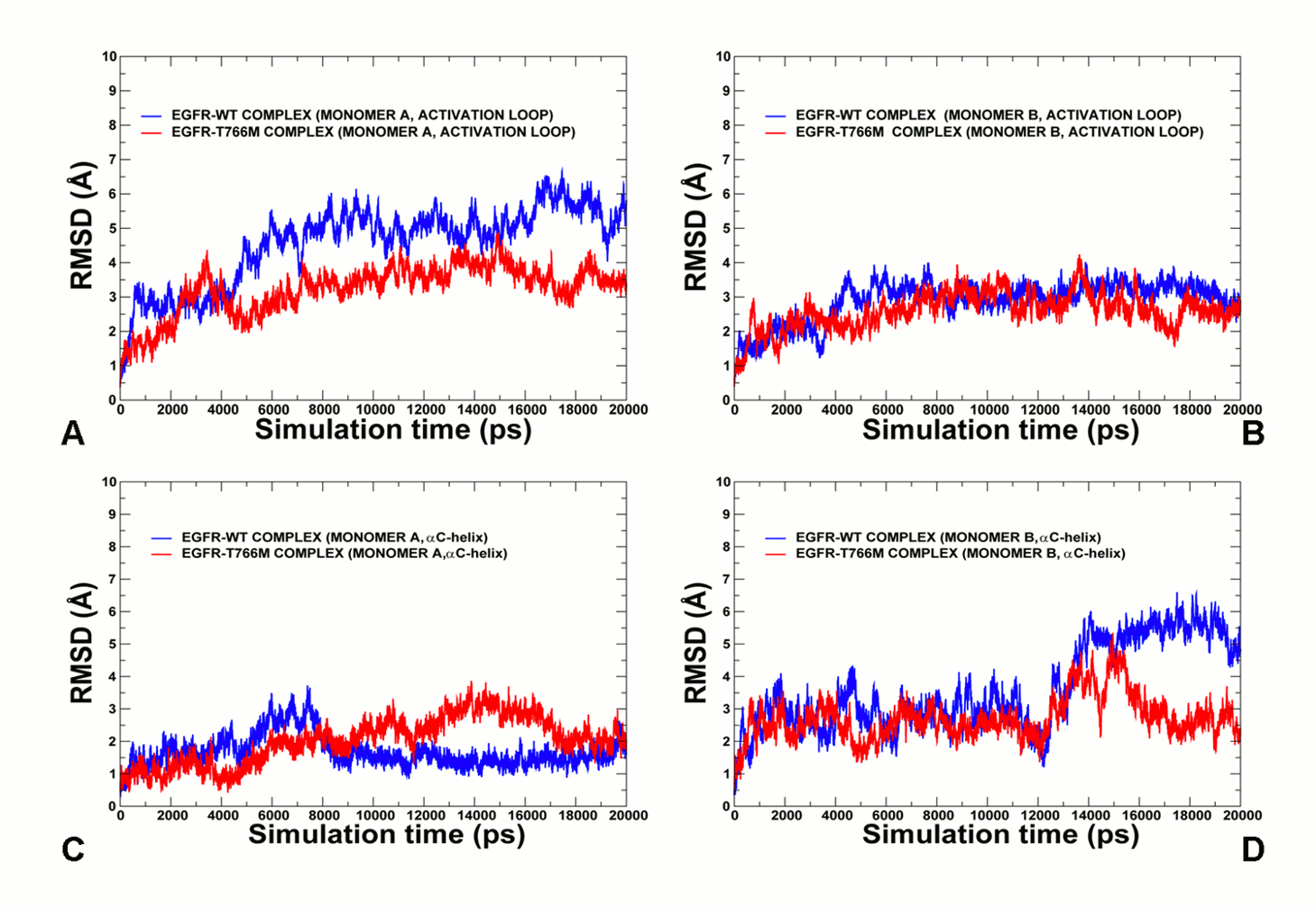

Supplement: Figure S3 — Protein flexibility of the EGFR catalytic core regulatory regions. Upper Panel: The RMSD fluctuations of Cα atoms in the activation loop of the monomer A (upper left panel A) and monomer B (upper right panel B) of an asymmetric EGFR dimer. EGFR-WT RMSD values shown in blue and EGFR-T766M shown in red. Lower Panel: The RMSD fluctuations of Cα atoms in the αC-helix of the monomer A (lower left panel C) and monomer B (lower right panel D) of an asymmetric EGFR dimer. EGFR-WT RMSD values shown in blue and EGFR-T766M shown in red. (TIF) [file pcbi.1002179.s003.tif]

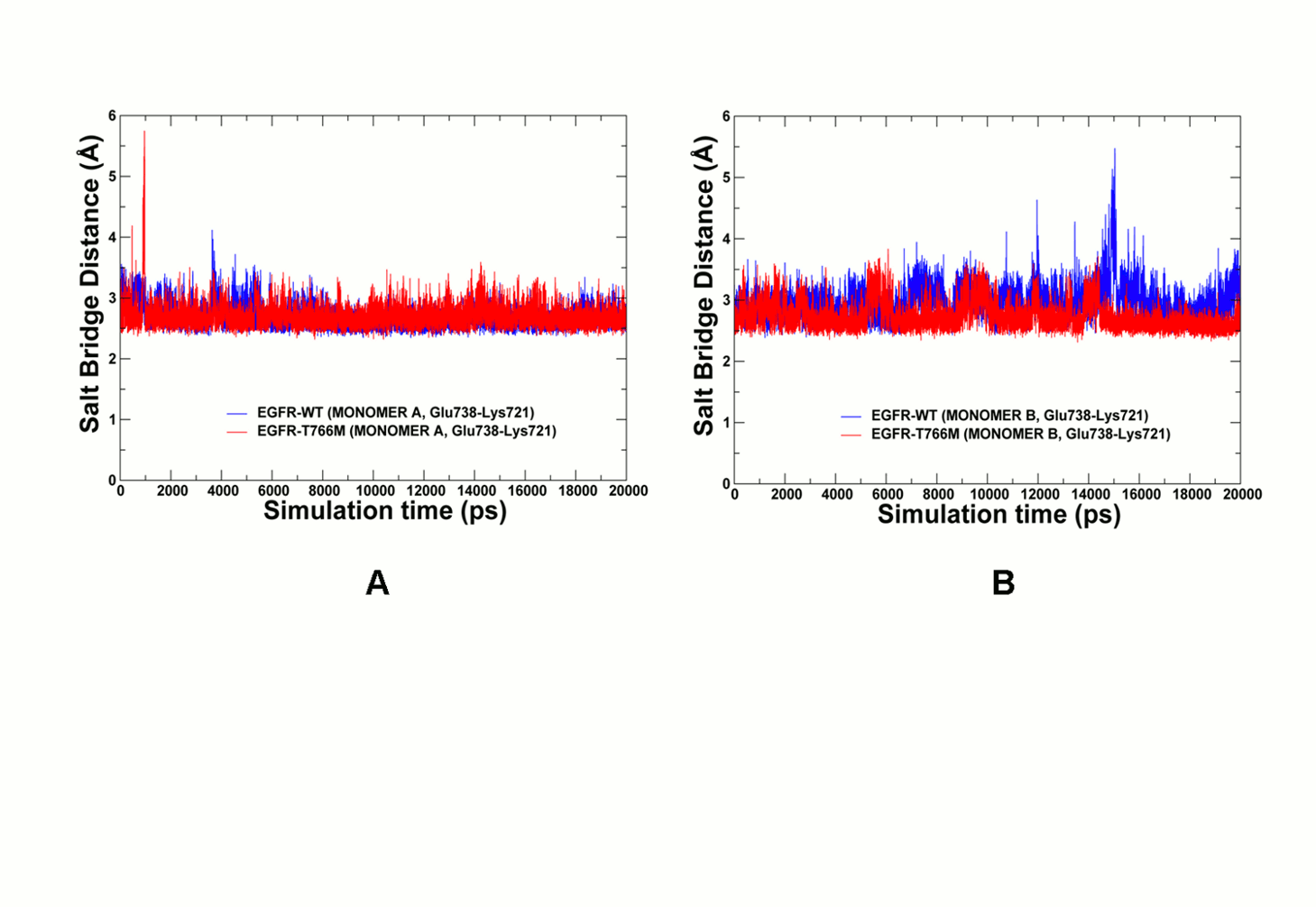

Supplement: Figure S4 — Time-dependent history of salt bridges from MD simulations of an asymmetric EGFR dimer. Thermal fluctuations of the salt bridge Glu738-Lys721 of the monomer A (left panel A) and monomer B (right panel B) of an asymmetric EGFR dimer. The flexibility profiles for EGFR-WT shown in blue and EGFR-T766M shown in red. (TIF) [file pcbi.1002179.s004.tif]

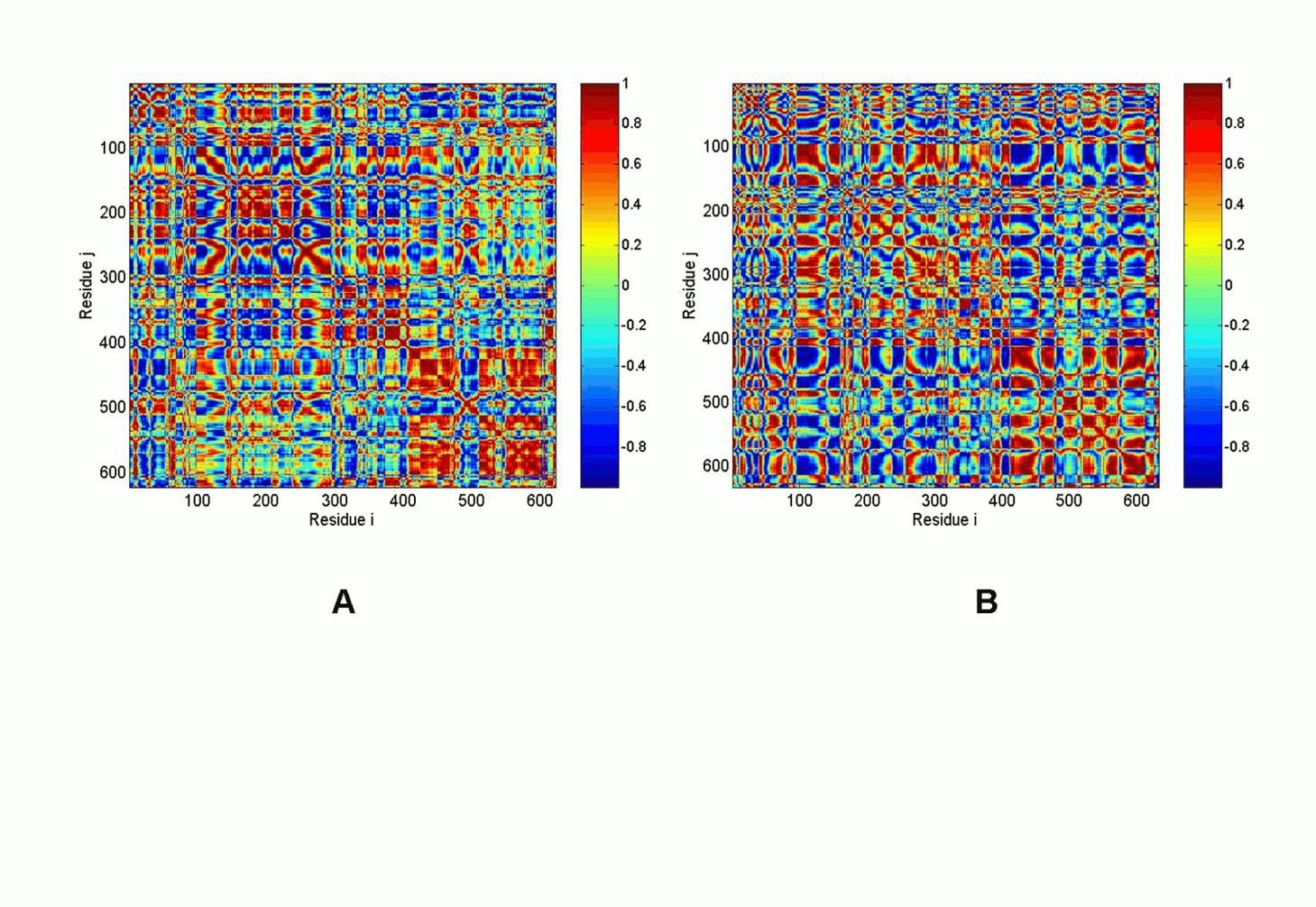

Supplement: Figure S5 — PCA Analysis of EGFR Dimers. The covariance matrix was calculated from 20 ns MD trajectories of the EGFR-WT asymmetric (A) and symmetric dimers (B). Translational and rotational degrees of freedom are eliminated and the average atomic coordinates are calculated using 500 frames from the 20 ns MD trajectories. MD trajectories were projected onto the main essential direction, corresponding to the largest eigenvector. Cross-correlations of residue-based fluctuations vary between +1 (fully correlated motion; fluctuation vectors in the same direction, colored in dark red) and −1 (fully anti-correlated motions; fluctuation vectors in the same direction, colored in dark blue). Here, the values above 0.5 are colored in dark red and the lower bound in the color bar indicates the value of the most anti-correlated pairs. (TIF) [file pcbi.1002179.s005.tif]

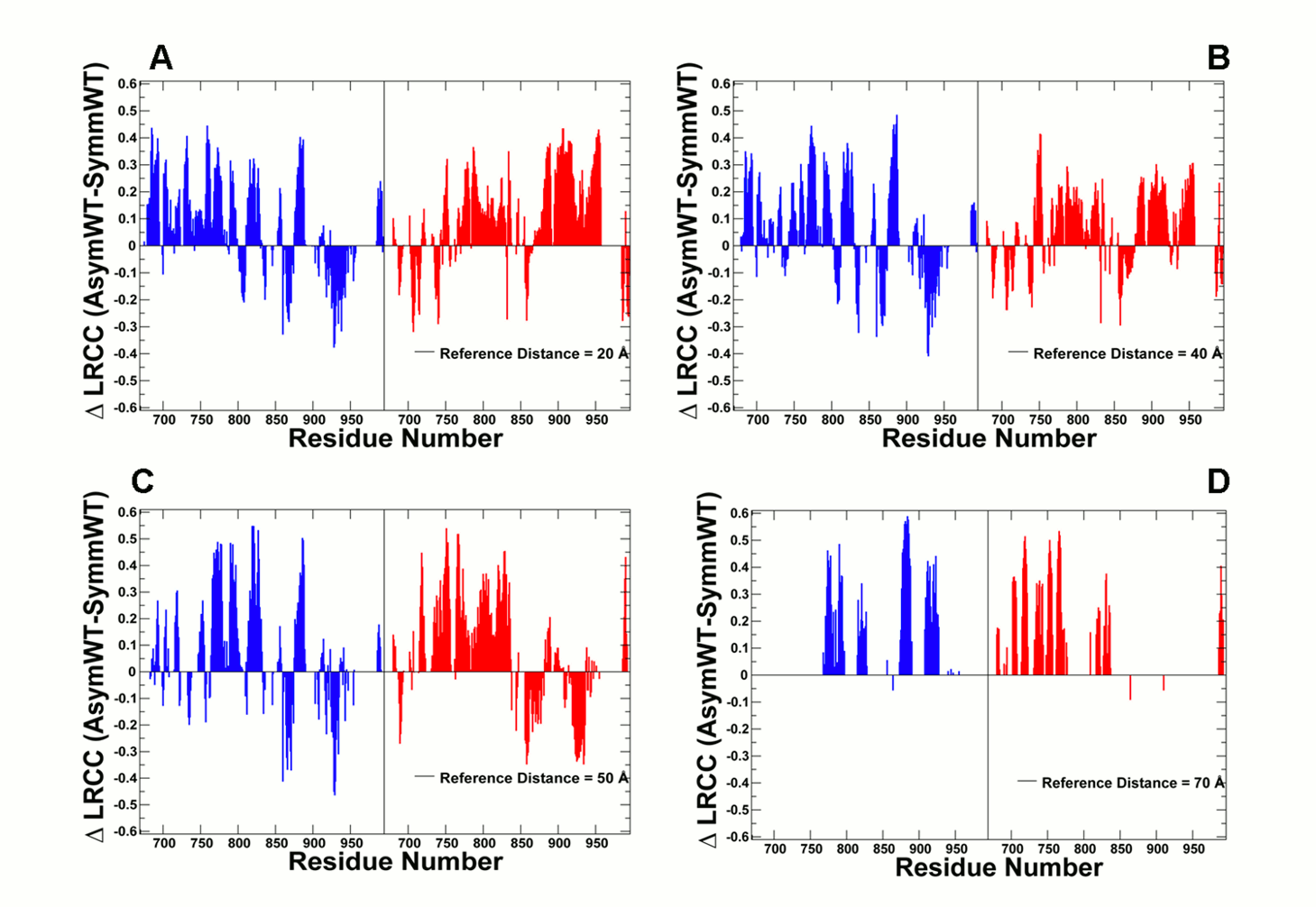

Supplement: Figure S6 — The Relative LRCC profiles for the EGFR Regulatory Dimers. The relative LRCC values between the asymmetric and symmetric EGFR-WT dimers computed at a range of reference communication distances: 20 Å (A), 40 Å (B), 50 Å (C), and 70 Å (D). Each bin refers to a residue and shows the fraction of residues that efficiently communicate with it () at distances greater the reference communication threshold of 30 Å. (TIF) [file pcbi.1002179.s006.tif]
